# YbiB: a novel interactor of the GTPase ObgE

**DOI:** 10.1093/nar/gkad127

**Published:** 2023-03-02

**Authors:** Babette Deckers, Silke Vercauteren, Veerke De Kock, Charlotte Martin, Tamas Lazar, Pauline Herpels, Liselot Dewachter, Natalie Verstraeten, Eveline Peeters, Steven Ballet, Jan Michiels, Christian Galicia, Wim Versées

**Affiliations:** VIB-VUB Center for Structural Biology, 1050 Brussels, Belgium; Structural Biology Brussels, Vrije Universiteit Brussel, 1050 Brussels, Belgium; VIB-KU Leuven Center for Microbiology, 3001 Leuven, Belgium; Centre of Microbial and Plant Genetics, KU Leuven, 3001 Leuven, Belgium; Research Group of Microbiology, Vrije Universiteit Brussel, 1050 Brussels, Belgium; Research Group of Organic Chemistry, Vrije Universiteit Brussel, 1050 Brussels, Belgium; VIB-VUB Center for Structural Biology, 1050 Brussels, Belgium; Structural Biology Brussels, Vrije Universiteit Brussel, 1050 Brussels, Belgium; VIB-KU Leuven Center for Microbiology, 3001 Leuven, Belgium; Centre of Microbial and Plant Genetics, KU Leuven, 3001 Leuven, Belgium; VIB-KU Leuven Center for Microbiology, 3001 Leuven, Belgium; Centre of Microbial and Plant Genetics, KU Leuven, 3001 Leuven, Belgium; VIB-KU Leuven Center for Microbiology, 3001 Leuven, Belgium; Centre of Microbial and Plant Genetics, KU Leuven, 3001 Leuven, Belgium; Research Group of Microbiology, Vrije Universiteit Brussel, 1050 Brussels, Belgium; Research Group of Organic Chemistry, Vrije Universiteit Brussel, 1050 Brussels, Belgium; VIB-KU Leuven Center for Microbiology, 3001 Leuven, Belgium; Centre of Microbial and Plant Genetics, KU Leuven, 3001 Leuven, Belgium; VIB-VUB Center for Structural Biology, 1050 Brussels, Belgium; Structural Biology Brussels, Vrije Universiteit Brussel, 1050 Brussels, Belgium; VIB-VUB Center for Structural Biology, 1050 Brussels, Belgium; Structural Biology Brussels, Vrije Universiteit Brussel, 1050 Brussels, Belgium

## Abstract

Obg is a widely conserved and essential GTPase in bacteria, which plays a central role in a large range of important cellular processes, such as ribosome biogenesis, DNA replication, cell division and bacterial persistence. Nevertheless, the exact function of Obg in these processes and the interactions it makes within the associated pathways remain largely unknown. Here, we identify the DNA-binding TrpD2 protein YbiB as an interactor of the *Escherichia coli* Obg (ObgE). We show that both proteins interact with high affinity in a peculiar biphasic fashion, and pinpoint the intrinsically disordered and highly negatively charged C-terminal domain of ObgE as a main driver for this interaction. Molecular docking and X-ray crystallography, together with site-directed mutagenesis, are used to map the binding site of this ObgE C-terminal domain within a highly positively charged groove on the surface of the YbiB homodimer. Correspondingly, ObgE efficiently inhibits the binding of DNA to YbiB, indicating that ObgE competes with DNA for binding in the positive clefts of YbiB. This study thus forms an important step for the further elucidation of the interactome and cellular role of the essential bacterial protein Obg.

## INTRODUCTION

Proteins of the Obg family belong to the TRAFAC class of P-loop GTPases. In general, P-loop GTPases or G-proteins are found in all domains of life where they act as central regulators in numerous cellular processes by alternating between an active GTP-bound state and an inactive GDP-bound state. The conversion of GTP to GDP is carried out by their characteristic G-domain, harboring five conserved sequence motifs responsible for nucleotide binding and hydrolysis (1–3). As the intrinsic GTP hydrolysis rate of P-loop GTPases is generally low, the reaction is often accelerated by GTPase activating proteins (GAPs), while guanine nucleotide exchange factors (GEFs) usually stimulate the exchange of GDP for GTP as nucleotide affinities are typically high in these GTPases (low nanomolar range) (4,5).

The Obg protein family is further divided in five subfamilies: YchF, Ygr210, Nog1, DRG and Obg. GTPases from the Obg subfamily are widely conserved among bacteria, and also occur in many eukaryotes (3,6). In bacteria, Obg proteins typically consist of an N-terminal glycine-rich domain, followed by the G-domain and a less conserved C-terminal domain. In Obg from *Escherichia coli* (ObgE), the latter is an intrinsically disordered region of about 50 amino acids, containing a high number of negatively charged amino acids (7–10). Similar to many other G-proteins, Obg displays a very low intrinsic GTP hydrolysis rate (6–8). However, while binding to the ribosome was reported to increase the GTPase activity (7,11), no ‘canonical’ GAP proteins have been assigned to Obg. Moreover, in contrast to ‘classical’ Ras-like G-proteins, Obg displays only a moderate nucleotide affinity (high nanomolar to low micromolar range) and correspondingly high nucleotide exchange rates, seemingly rendering regulation by GEF proteins obsolete (7,8,12–15). Finally, while G-proteins generally function as protein binding hubs to exert their function as cellular regulators, very little interactors of Obg have been firmly established so far (16,17).

A role of Obg in a wide variety of fundamental cellular processes has been proposed over the years (6). The Obg protein was first described in *Bacillus subtilis*, where it is important for sporulation initiation (18). Additionally, in *Streptomyces* Obg was found to be involved in the formation of aerial mycelium (19,20), indicating that Obg plays a role in the onset of morphological differentiation in some bacteria. A plethora of experimental data indicates that Obg also plays a role in ribosome biogenesis as a late-stage assembly factor for the large 50S ribosomal subunit, while it also prevents untimely association of the 30S subunit and sterically hinders premature association of tRNA to the ribosome (11,21,22). Besides this regulatory role in ribosome biogenesis, Obg has been implicated in DNA replication, chromosome segregation and cell division (23–29). Furthermore, studies in *E. coli* and *Vibrio harveyi* suggest that Obg confers protection against UV-induced DNA damage, most probably by directly or indirectly activating RecA-dependent DNA repair systems (30–32). The observation that Obg binds efficiently to the alarmone ppGpp also suggests a functional link between Obg and the stringent response (7,8,13,33). This is further corroborated by coprecipitation and two-hybrid assays demonstrating that ObgE interacts with SpoT, a ppGpp synthetase/hydrolase regulating the stringent response in *E. coli* (15,34). Finally, Obg was identified as a key regulator of bacterial persistence in *E. coli* and *Pseudomonas aeruginosa* (35,36). Bacterial persistence allows a small subset of bacteria within an isogenic population to withstand antibiotic treatment and is considered to be a major cause of chronic bacterial infections (37,38). Due to its conservation, functional versatility and its implication in bacterial persistence, Obg has been suggested as an attractive novel target for the development of antimicrobial compounds (39,40). Nevertheless, the exact role of Obg in the above-described processes and the interactions it makes within the associated pathways are still largely elusive. Upon searching for new ObgE interactors, we identified the DNA-binding TrpD2 protein YbiB.

The TrpD2 protein family is part of the phosphoribosyl transferase III superfamily and shares a common fold with the homologous anthranilate phosphoribosyltransferase (TrpD) and nucleoside phosphorylase class II (NP-II) families. Correspondingly, the X-ray crystal structure of the *E. coli* TrpD2 protein, referred to as YbiB, shows a head-to-head homodimeric architecture in which each monomer consists of an N-terminal α-helical domain and a C-terminal α/β domain. Despite their structural similarity, proteins of the TrpD2 family do not share the catalytic function of the TrpD nor the NP-II family. In contrast, the YbiB dimer displays two highly positively charged grooves on its surface with which it binds nucleic acids in a seemingly sequence-independent manner. Interestingly, in *E. coli*, the *ybiB* gene is part of a LexA-regulated operon, together with the *dinG* helicase gene. As LexA is the main suppressor of the SOS stress response, this suggests that YbiB may play a so far unknown role in the SOS stress response, which is activated upon DNA damage (41).

In this paper, we establish YbiB as a novel high-affinity interactor of ObgE. Biochemical experiments, together with computational docking and X-ray crystallography establish that the binding is mediated by interaction of the negatively charged tail of ObgE with the positively charged patches on the surface of YbiB. Accordingly, ObgE interferes with YbiB’s DNA-binding capacity.

## MATERIALS AND METHODS

The most important reagents, primers, peptides, plasmids and strains used in this paper are listed in Supplementary Table S1. In addition, all software programs and web servers used in this paper are listed in Supplementary Table S2.

### Identification of ObgE interaction partners by photoreactive crosslinking

To find ObgE interactors, an *in vivo* photocrosslinking approach was used by incorporating the photocrosslinker *p-*benzoyl-l-phenylalanine (*p*Bpa) in the expressed ObgE protein. The detailed procedure of this crosslinking experiment and the subsequent identification of interactors is given in the Supplementary Methods. In short, we used adaptations to the previously developed method by Chin *et al.* (42). Amber codons were introduced into pBAD/His A-*obgE* (35) and pBAD/His A-*obgE*_D246G_ (43) using the QuikChange Site-Directed Mutagenesis Kit (Stratagene) at positions corresponding to the ObgE amino acids W122, I250, Y269 and Y388. *p*Bpa was incorporated at these positions by expressing a tRNA/engineered tRNA synthase pair originating from *Methanocaldococcus jannashii* from the plasmid pSup-BpaRS-6TNR(D286R) (a gift from Peter Schultz, the Scripps Research Institute). After formation of the covalent crosslinks by irradiation with near-UV light, the ObgE interaction complexes were purified using Co^2+^ IDA affinity purification, and the interactors were identified using liquid chromatography coupled to tandem mass spectrometry (LC-MS/MS).

### Bacterial two-hybrid (B2H)

For validation of protein–protein interactions by bacterial two-hybrid, *obgE* and *ybiB* were amplified by PCR using genomic DNA of *E. coli* BW25113 as a template and primers SPI-12423 and SPI-12424 (*obgE*) or SPI-12671 and SPI-12672 (*ybiB*). These primers include XbaI and KpnI restriction sites, allowing easy introduction in pUT18, pUT18C, pKT25 and pNT25 (Euromedex). Postulated protein interactions were validated using the BACTH System Kit (Euromedex). This system is based on the interaction-mediated fusion of two complementary fragments, T25 and T18, which restores adenylate cyclase activity in a *cya-*deficient *E. coli* reporter strain, *i.e*. RH785. A functional adenylate cyclase catalyzes the conversion of ATP to cyclic adenosine monophosphate (cAMP), which in turn binds to the catabolite activator protein (CAP). Together, cAMP and CAP trigger transcriptional activation of the *lacZ* reporter gene.

For all strains, the two plasmids carrying the T25 and the T18 hybrid genes were consecutively transferred to RH785 competent cells. Cultures were grown overnight at 37°C in LB supplemented with 100 μg/ml ampicillin and 50 μg/ml kanamycin, shaking at 200 rpm. The overnight cultures were then diluted 1:100 in 5 ml LB medium supplemented with 0.5 mM β-d-1-thiogalactopyranoside (IPTG), ampicillin and kanamycin. After 16 h, 135 μl of each culture was transferred to a 96-well plate, and the initial OD_595_ value was measured. Subsequently, 10 μl of each sample was transferred to a new 96-well plate. β-galactosidase activity of the activated *lacZ* was detected in a quantitative detection assay measuring the hydrolysis of ortho-nitrophenyl-β-d-galactoside (ONPG) to the yellow substrate ortho-nitrophenol (ONP). To this end, 90 μl of substrate buffer (50 mM PBS, 10 mM β-mercapto-ethanol, 0.1% Triton X-100, 0.1% natrium lauroylsarcosine, 1 mM Na_2_EDTA and 2.5 mM ONPG in Milli-Q water) was added to each sample and the plates were incubated at 30°C for 30 min. To stop the reaction, 35 μl of stop buffer (1 M Na_2_CO_3_ in Milli-Q water) was added to the wells and the OD_595_ and OD_415_ were measured with a spectrophotometer. Miller units (MU) were calculated as follows:


$$\begin{eqnarray*} && MU =\nonumber\\ && \frac{{\left( {O{D}_{415,sample} - O{D}_{415,blank}} \right) - 1.75*\left( {O{D}_{595,sample} - O{D}_{595,blank}} \right)}}{{\left( {O{D}_{595,cell} - O{D}_{595,cell,blank}} \right)*t}}\ *1000*13.5\end{eqnarray*}$$

With *OD_x,sample_* referring to the optical density at x nm after incubation and stopping the reaction. The underscore ‘*blank*’ represents blank LB samples and ‘*cell*’ represents the 135 μl culture sample before incubation. ‘*t*’ indicates the time of incubation in minutes (44).

### Persistence assay

The construction of pBAD/His A-*obgE* and pBAD33Gm has previously been described (35,43,45). pBAD33Gm-*ybiB* was constructed by Gibson Assembly using primers SPI-13850, SPI-13851, SPI-13852 and SPI-13853. A clean deletion mutant of *ybiB* in BW25113 was constructed as described by Datsenko *et al.* (46) using primers SPI-12822 and SPI-12823. Single-gene knockout strains were retrieved from the Keio collection (47). The pBAD33Gm, pBAD33Gm-*ybiB*, pBAD/His A and pBAD/His A-*obgE* plasmids were all separately transferred to BW25113 competent cells. In addition, the pBAD/His A and pBAD/His A-*obgE* plasmids were transferred to BW25113 Δ*ybiB* competent cells. For phenotypic analyses, cultures were grown overnight in LB supplemented with 100 μg/ml ampicillin (pBAD/His A) or 25 μg/ml gentamicin (pBAD33Gm) at 37°C, shaking at 200 rpm. The overnight cultures were diluted 1:100 in 5 ml fresh LB medium supplemented with the appropriate antibiotics and l-(+)-arabinose (0.2% w/v). After 16 h of incubation, 1 ml of culture was treated with 5 μg/ml ofloxacin, and, as a control treatment, sterile water was added to another 1 ml aliquot. Both aliquots were incubated for 5 h, after which the number of surviving cells was determined by plate counting. Based on the plate counts, persister fractions were determined by comparing the treated with the untreated cultures. These fractions were log_10_ transformed after which the differences between the fractions were statistically compared using an unpaired t-test with Welch's correction.

### Cloning, protein expression and protein purification

Expression and purification of the N-terminally His_6_-tagged ObgE protein (ObgE) and its C-terminally truncated variant (ObgE_1–340_) were performed as previously described with minor modifications (7). In short, the open reading frames coding for these proteins were cloned in a pET28a vector (Novagen) and proteins were expressed in *E. coli* BL21 (DE3) pLysS cells. The proteins were subsequently purified on a HisTrap FF column (5 ml, Cytiva) and nucleotide-free protein was obtained using alkaline phosphatase treatment (7). To remove the alkaline phosphatase after treatment, either a second passage over a HisTrap FF column or an anion exchange on a HiTrap Q HP column (5 ml, Cytiva) was performed. For the latter, the protein was dialyzed against 18 mM PIPES pH 6.5, 150 mM NaCl, 5% glycerol, 2 mM DTT, loaded on the column and, after extensive washing, eluted using 18 mM PIPES pH 6.5, 1 M NaCl, 5% glycerol, 2 mM DTT. Finally, a last purification step consisted of a size exclusion chromatography on a Superdex 75 26/60 or 16/60 column (GE Healthcare) equilibrated in 20 mM HEPES pH 7.5, 150 mM NaCl, 5 mM MgCl_2_, 2 mM DTT and 5% glycerol. The purified ObgE protein constructs were flash frozen in liquid nitrogen and stored at -80°C. For performing pull-down experiments, a C-terminally Twin-Strep-tagged ObgE protein construct (ObgE-Strep) was generated as well, using the InFusion cloning method. To do so, a pET22b vector carrying an ORF coding for C-terminally Strep-tagged ObgE was used as a template (7). To replace the single with the double C-terminal Strep-tag, a double-stranded DNA fragment harboring part of the ObgE C-terminus fused to a Twin-Strep-tag (gBlocks™ Gene Fragments, Integrated DNA Technologies) was amplified through PCR and subsequently cloned within the EcoRI and XhoI sites of the template pET22b vector using the InFusion^®^ HD cloning kit (Takara Bio). Purification of ObgE-Strep was performed analogously to above with the exception that the purification on the HisTrap column was replaced by purification on Strep-Tactin^®^ Sepharose^®^ resin (iba life sciences), using 20 mM HEPES pH 7.5, 300 mM NaCl, 5 mM MgCl_2_, 5 mM β-mercapto-ethanol and 5% glycerol for washing the column after loading the protein, and using the same buffer supplemented with 2.5 mM desthiobiotin for elution.

The open reading frame coding for the full-length *E. coli* YbiB protein was amplified by PCR using *E. coli* BW25113 genomic DNA as template. The PCR product was subsequently cloned within the NheI and EcoRI restriction sites of a pET28a vector that contains the sequence for an N-terminal His_6_-tag followed by a thrombin cleavage site. The YbiB mutants (Arg92Glu and Arg123Glu) were generated by use of the QuikChange Site-Directed Mutagenesis Kit (Stratagene). The YbiB protein constructs were all expressed in *E. coli* BL21 (DE3) pLysS cells. Cells were grown in Terrific Broth (TB) medium supplemented with 25 μg/ml kanamycin at 37°C and shaking at 120 rpm. After reaching an OD_600 nm_ of 0.7, the cultures were induced with 1 mM IPTG and further grown overnight at 25°C and 120 rpm. After harvesting the cells by centrifugation, the cell pellets were resuspended in lysis buffer (20 mM HEPES pH 7.5, 300 mM NaCl, 5 mM β-mercaptoethanol, 10 mM imidazole) containing 0.1 mg/ml AEBSF, 1μg/ml leupeptin and 50 μg/ml DNase. The cells were then lyzed using a cell disruptor system (Constant Systems) after which the cell lysates were cleared by centrifugation. The cleared lysates were loaded onto a pre-equilibrated HisTrap FF column (5 ml, Cytiva). The column was extensively washed with 20 mM HEPES pH 7.5, 1 M NaCl, 5 mM MgCl_2_, 10 mM imidazole and 5 mM β-mercaptoethanol and proteins were eluted using a linear gradient of elution buffer containing 20 mM HEPES pH 7.5, 300 mM NaCl, 5 mM MgCl_2_ 500 mM imidazole and 5 mM β-mercaptoethanol. The proteins were then further purified through size exclusion chromatography on a Superdex 200 26/60 column (GE Healthcare) equilibrated in 20 mM HEPES pH 7.5, 300 mM NaCl, 5 mM MgCl_2_, 1 mM DTT. The purified YbiB proteins were flash frozen in liquid nitrogen and stored at −80°C.

### Synthesis of ObgE-derived peptides

Peptides were synthesized using Fmoc-based solid phase peptide synthesis (SPPS) on a microwave-assisted peptide synthesizer (CEM Liberty Lite). All preloaded Wang resins (Fmoc-Arg(Pbf)-Wang, Fmoc-Asp(tBu)-Wang, Fmoc-Glu(tBu)-Wang, Fmoc-Gln(Trt)-Wang) and the Rink Amide resin were purchased from Advanced Chemtech. The synthesis was performed on a 0.1 mmol scale using preloaded Wang resin or Rink Amide resin depending on the desired C-terminal functionality of the peptide, being a carboxylic acid or carboxamide. Fmoc deprotection was performed at 90°C for 3 min using a solution of 20% 4-methylpiperidine in *N,N*-dimethyl formamide (DMF) during the entire synthesis. Each coupling was done using 5 equivalents of Fmoc-protected amino acid (Chem-Impex), with 0.5 M *N,N’*‐diisopropylcarbodiimide (DIC) and 1 M ethyl cyano(hydroxyimino)acetate (Oxyma) as coupling mixture. N-terminal acetylation was done manually using 10 equivalents of acetic anhydride and 5 equivalents of diisopropylethylamine (DIPEA) during 1 hour in DMF. After completion of the sequence, the resin was washed several times with dichloromethane (DCM), followed by treatment with a cleavage cocktail solution consisting of 90% trifluoroacetic acid (TFA), 5% triisopropylsilane and 5% distilled water during 4 hours. After freeze-drying, crude peptides were obtained and purified using preparative HPLC. More specifically, a Gilson HPLC system, equipped with Gilson 322 pumps and a Supelco Discovery^®^ BIO Wide Pore C18 column (25 cm × 21.2 mm, 10 μm), was used. Crude peptides were dissolved in dimethyl sulfoxide (DMSO) and purified using H_2_O-acetonitrile–0.1% TFA as mobile phase. Finally, fractions were collected, of which the purities were assessed by analytical RP-HPLC. Finally, the pure fractions were combined and lyophilized to obtain the purified peptide as a powder (TFA salt) with high purity (>97%). A summary of the peptide sequences and their characteristics can be found in Supplementary Table S3.

### Pull-down experiments

To obtain a homogeneous nucleotide load, the nucleotide-free ObgE-Strep protein was incubated (on ice) for 1 h with 1 mM of the nucleotide before starting the experiment. ObgE-strep was mixed with the His_6_-tagged YbiB protein in a 1:3 molar ratio (final concentrations of 10 and 30 μM, respectively) in binding buffer (20 mM HEPES pH 7.5, 300 mM NaCl, 5 mM MgCl_2_, 5 mM β- mercaptoethanol, 5% glycerol), either with or without 100 μM of the corresponding nucleotide. Samples containing either only ObgE-strep or YbiB were diluted in binding buffer to the corresponding concentration and served as controls. The samples were incubated on ice for 1 h and an ‘input’ sample was taken before incubating with Strep-Tactin^®^ Sepharose^®^ resin (iba lifesciences) on ice for 30 min. Each sample was centrifuged at 1000 × g for 1 min at 4°C and the supernatant served as unbound fraction (‘flow-through’). Subsequently, the beads were washed six times with 120 μl binding buffer, collecting the supernatant as wash fractions. The proteins were eluted by incubating the beads on ice for 5 min with 100 μl elution buffer (binding buffer containing 2.5 mM desthiobiotin), followed by centrifugation at 1000 × g for 1 min at 4°C. The supernatant was kept as elution fraction. Finally, the different fractions (input, flow-through, wash and elution) were analyzed on SDS-PAGE.

### Isothermal titration calorimetry (ITC)

All ITC measurements were performed using a MicroCal iTC200 system (GE Healthcare) at 25°C and a buffer containing 20 mM HEPES pH 7.5, 150 mM NaCl, 5 mM MgCl_2_ and 1 mM β-mercaptoethanol. The ObgE protein constructs and ObgE-derived peptides (Supplementary Table S3) were loaded into the sample cell at concentrations ranging from 50 to 75 μM, while YbiB was loaded into the syringe at concentrations between 500 μM and 1.4 mM. A reference power of 10 μcal/s was used, and 18 injections of 2 μl, preceded by a test injection of 0.4 μl, were performed at 180 s intervals while stirring at 750 rpm. An initial delay of 60 s was included before the start of each run. The MicroCal PEAQ-ITC Analysis software (version 1.30, Malvern Panalytical) was used for data integration and to fit the resulting binding isotherms to the appropriate binding model provided by the software, with the ‘Ligand in cell’ function activated. The ‘two sets of sites’ model that was used to fit some of the binding isotherms assumes the presence of two different binding sites that are independent of each other. The equations that describe this model are provided in the Malvern Microcal iTC200 user manual (https://www.malvernpanalytical.com/en/learn/knowledge-center/user-manuals/MAN0560EN).

### Multi-angle light scattering (MALS), mass photometry and small angle X-ray scattering (SAXS)

For SEC-MALS, a Superdex 200 5/150 GL increase column (Cytiva) was coupled to a HPLC Alliance system (Waters) equipped with a 2998 PDA detector (Waters), a TREOS II MALS detector (Wyatt Technology) and a RI-501 refractive index detector (Shodex). Samples of ObgE and YbiB separately were prepared at a final concentration of 1.5 mg/ml. For the YbiB–ObgE complex, the proteins were incubated on ice in a 1:3 molar ratio (final concentrations of 20 and 60 μM ,respectively) before injection. For each sample, 20 μl was injected and 20 mM HEPES pH 7.5, 150 mM NaCl, 5 mM MgCl_2_, 5% glycerol and 2 mM DTT was used as running buffer at a flow rate of 0.2 ml/min. The Astra 7.3.0 software (Wyatt Technology) was used to analyze the data. A BSA sample (1 mg/ml) was used to normalize and align the signals of the different detectors and to account for any band broadening effects before further analyzing the other runs. A d*n*/d*c* value of 0.1850 ml/g was used.

Mass photometry measurements were performed for YbiB in PBS buffer at concentrations of 0.0125 and 0.005 mg/ml using the Refeyn OneMP instrument. Data was collected using the Refeyn AcquireMP software (version 2022 R1) and analyzed in the Refeyn DiscoverMP software (version 2022 R1). Molecular weights were determined using the NativeMark™ Unstained Protein Standard (ThermoFischer Scientific) as reference.

SEC-SAXS was performed using pre-formed YbiB-ObgE complex, obtained by incubating a mixture of YbiB and ObgE in a 1:3 molar ratio in buffer containing 20 mM HEPES pH 7.5, 150 mM NaCl, 5 mM MgCl_2_, 2 mM DTT and 5% glycerol. After 1 h incubation on ice, the sample was run through a pre-equilibrated Superdex 200 10/300 increase column (GE Healthcare) to isolate the complex. The YbiB-ObgE complex was subsequently concentrated by use of a 10 kDa cut-off centrifugal concentrator (Vivaspin^®^, Sartorius) to a concentration of 7.6 mg/ml. SAXS data were collected at the P12 beamline operated by EMBL Hamburg at the PETRA III storage ring (DESY, Hamburg, Germany) (48). 50 μl of the sample was loaded on a pre-equilibrated Superdex 200 10/300 column at a flow rate of 0.6 ml/min. The radial averaging of the collected SAXS data was performed using the IM2DAT tool within the SASFLOW program (49). After subtraction of the background signal by the use of CHROMIXS (50), the averaged data, corresponding to the peak of interest, were further analyzed using the ATSAS software package (51). The Guinier, Kratky and pair distance distribution plots were obtained using PRIMUS (52), while the Bayesian inference method was used to determine the molar mass of the complex (53) (Supplementary Table S4).

### Circular dichroism (CD) spectroscopy

For CD measurements, a MOS-500 CD spectrometer (BioLogic) was used. Spectra were recorded using a 1 mm cuvette and a slit bandwidth of 1 nm. All measurements were performed at 25°C in a buffer containing 20 mM Tris pH 7.5, 150 mM NaCl and 5 mM MgCl_2_. For each sample, three repeat measurements were collected. The spectra of the YbiB and ObgE peptide1 (SBL-OBGE-01, Supplementary Table S3) samples were measured at a concentration of 13.3 μM, while the complex sample was prepared by mixing 13.3 μM YbiB with 13.3 μM peptide1. The final CD spectra (expressed in ellipticity θ) were obtained after subtraction of the buffer spectrum.

### Modeling and peptide docking

AlphaFold-Multimer (54) was used to model the complex between a YbiB dimer and two copies of the C-terminal ObgE peptide ^361^LEEIAEEDDEDWDDDWDEDDEE^382^, which covers the whole 17-residue-long (amino acids 366–382) palindrome of the ObgE C-terminus. The publicly available code of ColabFold (55), installed on a local server, was used to run AlphaFold-Multimer with default parameters. Amber relaxation was used during refinement. The number of recycles was set to 9, while the number of output models was set to 5.

The motif-based ClusPro-PeptiDock peptide docking protocol was used for unbiased global peptide docking (56). A model of YbiB containing all loop regions was taken from the AlphaFold Protein Structure Database (57,58) and converted into the biological dimer by superposition on the available experimental YbiB structure (PDB: 4MUO) (41). This model was provided as receptor structure to ClusPro. The palindromic motif pattern ‘EXWXXXWXE’ was used as peptide motif input sequence, which resulted in 229 hits from the PDB. Using the balanced scoring scheme, ClusPro-PeptiDock produced 53 models for YbiB in complex with the ObgE peptide (^370^EDWDDDWDE^378^). These models represent clusters of binding poses, with the most populated ten clusters sampled 498, 378, 130, 130, 121, 117, 114, 106, 106 and 103 times, respectively. This renders clusters 1 and 2 as the most reliable ones, which are symmetrical solutions binding different subunits of YbiB on the same interface in the same orientation.

The same unbiased global peptide docking was repeated using CABS-dock that can model much longer flexible peptides without making use of templates from the PDB (59,60). Similarly, the dimeric YbiB AlphaFold model was used as receptor structure while an ObgE peptide sequence (^361^LEEIAEEDDEDWDDDWDEDDEE^382^) that contains the whole 17-residue-long (amino acids 366 to 382) palindrome of the ObgE C-terminus was used. Simulation length was doubled from the default 50–100 Monte Carlo simulation cycles as recommended by the authors of the tool. The ten CABS-dock output models for the YbiB-ObgE protein-peptide complex represent clusters of binding poses with clusters 1 to 10 being sampled 206, 177, 30, 198, 34, 32, 119, 20, 43 and 52 times, respectively. Therefore, representative models of clusters 1, 2 and 4 are considered to be the ones of highest confidence.

### X-ray crystallography

In an attempt to obtain crystals of YbiB in complex with either the ObgE C-terminal peptide1 (SBL-OBGE-01, Supplementary Table S3) or peptide4 (SBL-OBGE-03, Supplementary Table S3) YbiB was incubated with a 1.5× molar excess of the peptide and concentrated to a final complex concentration of 20 or 15 mg/ml, respectively. Several commercial crystallization screens were used via the sitting-drop vapor-diffusion method at 20°C. Crystals resulting from the mixture with peptide1 were obtained in 15% w/v PEG 3350 and 0.1 M MES pH 6.2. Since these crystals turned out to not contain the peptide, the corresponding structure is hereafter referred to as ‘YbiB-apo’. For the YbiB-peptide4 complex, crystals were obtained in 20% w/v PEG6000 and 0.1 M citrate pH 5. The crystals were briefly soaked in a cryo-solution of mother liquor supplemented with 25% glycerol before flash-freezing them in liquid nitrogen. Diffraction data were collected at 100 K at the PROXIMA-1 beamline of the SOLEIL synchrotron (λ = 0.97856) using an Eiger-X 16M detector (Dectris Ltd) (61). Data indexing, integrating and scaling was performed using autoPROC (Global Phasing Limited) (62), including the programs XDS, Truncate, Aimless and STARANISO for anisotropic correction (Supplementary Figure S1) (Tickle *et al.*, 2018, https://staraniso.globalphasing.org/cgi-bin/staraniso.cgi).

Structures were solved by molecular replacement (Phaser (61) in Phenix (62)) using pdb 4MUO as model for the YbiB-apo structure and our refined apo structure as model for the YbiB-peptide4 structure. Both structural models were iteratively improved by cycles of manual building in Coot (63) alternated with refinement using Phenix.Refine (64), including TLS refinement during the last rounds (65). Analysis of the YbiB-peptide4 dataset revealed the presence of extra density that could be accounted for by a part of peptide4 (^375^DWDEDD^380^). This peptide segment was manually built in Coot and included early on in the refinement process. MolProbity (66) was used for structure validation. For the YbiB-peptide4 dataset, a composite omit map was generated, using Phenix.composite_omit_map in the simple mode. Data collection and refinement statistics are given in Supplementary Table S5. All structural figures were made using PyMOL (version 2.3.2) (The PyMol Molecular Graphic System, version 2.3.2 Schrödinger, LLC, https://pymol.org/2/). The ConSurf web server, with default parameters, was used to calculate the degree of residue conservation for YbiB (67,68). Structure factors and coordinates for the YbiB-apo and YbiB-peptide4 datasets have been submitted to the Protein Data Bank under accession codes 8BFR and 8BFT, respectively.

### Steady-state kinetics

The ObgE-catalyzed GTP hydrolysis was monitored over time in absence and presence of YbiB. Therefore, 0.5 μM ObgE was incubated on ice for 15 min either without or with 5 μM YbiB before adding different concentrations of GTP ranging from 2.5 to 100 μM. All measurements were performed in a buffer containing 20 mM HEPES pH 7.5, 150 mM NaCl, 5 mM MgCl_2_ and 2 mM DTT. The samples were placed at 25°C, while 40 μl aliquots were taken at different time points (0, 1, 2, 3 and 4 h). To stop the reaction these aliquots were immediately flash frozen and subsequently boiled for 3 min at 95°C to precipitate the proteins. To measure the amount of GDP formed at each time point, each aliquot was analyzed on a Kinetex 2.6 μm C18 100 Å 100 × 4.6 mm column (Phenomenex) coupled to an HPLC Alliance system (Waters) and eluted using 100 mM KH_2_PO_4_ (pH 6.4), 10 mM tetra-*n*-butylammonium bromide and 7.5% acetonitrile as a mobile phase. Nucleotides were detected at 254 nm. A GDP standard was used to convert the peak areas into GDP concentrations, and these concentrations were plotted in function of time to derive the initial rate from the slope of the linear trendline. Finally, a Michaelis–Menten curve was obtained by plotting the initial rates against the corresponding GTP concentration. All measurements were performed in triplicate. By fitting the final curves on the Michaelis-Menten equation, using Graphpad Prism (version 9.3.1), *K*_M_- and *k*_cat_-values (± SEM) were obtained.

### Electrophoretic mobility shift assays

The electrophoretic mobility shift assays (EMSAs) were performed using a 58 bp single stranded DNA probe (Supplementary Table S1) (41). A radioactive labeling reaction was performed by incubating the DNA (at a concentration of 10 pmol/μl) with 1 μl T4 polynucleotide kinase (Thermo Scientific) and 5 μl [γ-^32^P]-ATP (Perkin Elmer) for 40 min at 37°C in 1× kinase buffer (Thermo Scientific) in a total volume of 30 μl. The labeling reaction was quenched by placing the sample at 60°C for 10 min. The labeled DNA probe was purified from a 6% polyacrylamide gel run in 1× TBE buffer at room temperature. To perform the EMSAs, the ^32^P-labeled probe was incubated with different concentrations of YbiB (WT or mutant) for 30 min at 37°C in 20 mM HEPES pH 7.5, 150 mM NaCl, 5 mM MgCl_2_, 2 mM DTT, 5% glycerol. The samples were analyzed on a 6% polyacrylamide gel in 1× TBE buffer at room temperature. The gels were exposed overnight to a storage phosphor screen (Cytiva), which was then imaged using the Personal Molecular Imager™ (PMI™) system (Bio-Rad). For the EMSAs performed in presence of ObgE, YbiB was first incubated on ice for 30 min with different molar excesses of ObgE, before incubation with the DNA at 37°C. To determine apparent *K*_D_-values, the mean density of the unbound DNA bands on each EMSA were determined by use of the ImageJ software (69). After subtraction of the mean density of the background, the fraction of unbound DNA was determined. Based on the unbound fraction, the fraction of bound DNA was calculated and plotted against the protein concentration. This plot was fitted to the Hill equation, using Graphpad Prism (version 9.3.1), to obtain the apparent *K*_D_ ± fitting error.

## RESULTS

### Identification of YbiB as a novel persistence-related interaction partner of ObgE

Photocrosslinking was used to find novel interactors of ObgE (42). This technique relies on the incorporation of the photocrosslinker *p-*benzoyl-L-phenylalanine (*p*Bpa) on the surface of a protein of interest. Upon irradiation of the cells with near-UV light, *p*Bpa generates highly reactive intermediates that react with adjacent molecules (within a radius of 3 Å), resulting in direct covalent binding. High-resolution liquid chromatography coupled to tandem mass spectrometry (LC-MS/MS) can subsequently be used to identify the interaction complexes.

In this work, each of the ObgE residues W122, I250, Y269 and Y388 were individually replaced by the *p*Bpa photocrosslinker, using previously described procedures (42). These four residues are located on the surface of ObgE and are spread over its three domains. Importantly, replacement of aromatic (W, Y) or bulky (I) amino acids by *p*Bpa is expected to have minimal impact on protein folding, and successful formation of full-length ObgE proteins was confirmed by western blotting. Moreover, we confirmed that incorporation of *p*Bpa did not affect growth or persistence. Since we were initially mainly interested in interaction partners playing a role in persistence, *p*Bpa was introduced at the described positions in both the wild-type ObgE protein and in ObgE_D246G_, a mutant deprived of its persistence function (43). Excluding hits that interact with ObgE_D246G_ in the analysis allowed us to obtain 44 putative ObgE interactors with a potential role in persistence.

From this list, YbiB was selected as one of the priority hits for further investigation since it has previously been suggested to play a role in the SOS response (41), which is activated upon DNA damage and implicated in persistence (summarized in (70)). The postulated interaction between ObgE and YbiB was confirmed by a quantitative B2H assay, which is based on the interaction-mediated fusion of two complementary fragments, T25 and T18, thereby restoring adenylate cyclase activity in a *cya-*deficient *E. coli* reporter strain. As a positive control, the leucine zipper motifs of the yeast protein GCN4As fused to T25 and T18 (pKT25-*zip*, pUT18-*zip*) were included, demonstrating a strong interaction (71). In contrast, no interaction was observed between the T25 and T18 fragments alone. Since fusions in hybrid proteins might sterically hinder interactions, potential interactions between ObgE and YbiB were verified in eight different orientations. A positive signal was detected for the Obg–YbiB interaction in three out of eight orientations (*P* < 0.01) (Supplementary Figure S2).

**Figure 1.**
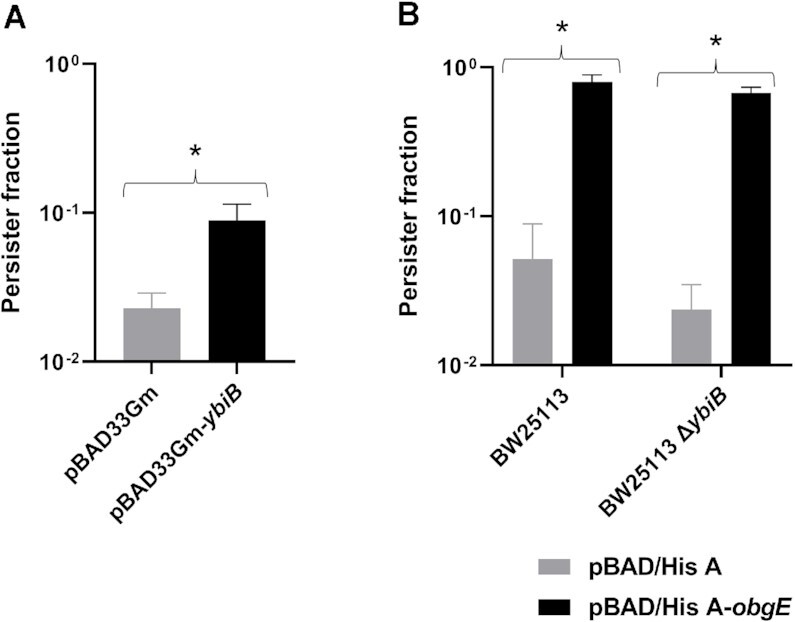
YbiB is involved in persistence. (**A**) Persister fractions after ofloxacin treatment of BW25113 containing either pBAD33Gm or pBAD33Gm-*ybiB*. Means ± SEM are presented (*n* = 13). *: *P*-value < 0.01. (**B**) Persister fractions after ofloxacin treatment of BW25113 and BW25113 Δ*ybiB* containing either pBAD/HisA or pBAD/HisA-*obgE*. Means ± SEM are presented (*n* = 7). *: *P*-value < 0.01.

Interestingly, overexpression of *ybiB* in a BW25113 *E. coli* strain increases the persister fraction, suggesting that YbiB is involved in bacterial persistence (Figure 1A). However, deletion of *ybiB* does not affect persistence significantly (grey bars in Figure 1B). While both ObgE and YbiB overexpression result in an increased persister fraction, YbiB and ObgE do not seem to act in serial fashion in the same pathway since ObgE overexpression in a *ybiB* knock-out strain still leads to an increased persister fraction (Figure 1B).

### ObgE and YbiB interact with high affinity

**Figure 2.**
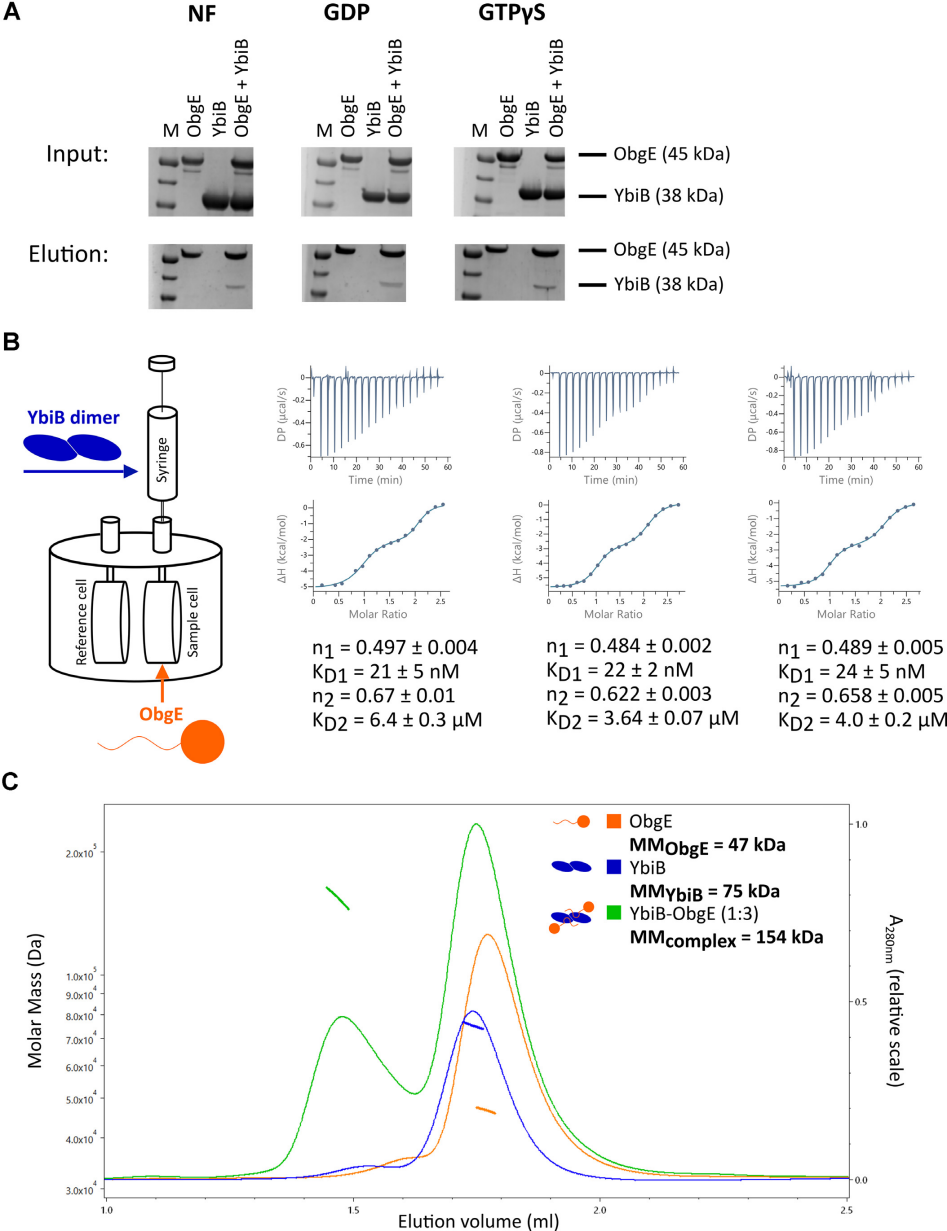
ObgE and YbiB interact with high affinity. (**A**) *In vitro* pull-down assays performed with purified ObgE (C-terminally Twin-Strep-tagged) and YbiB (N-terminally His_6_-tagged) using Strep-Tactin beads. The Strep-Tactin beads were incubated with ObgE alone, YbiB alone or a mixture of both proteins. Proteins were eluted with buffer containing desthiobiotin. The experiment was performed for different nucleotide states of ObgE (nucleotide free (NF), GDP-bound and GTPγS-bound). M: Molecular mass marker (PageRuler™ Prestained protein ladder, ThermoFischer Scientific). (**B**) ITC measurement to assess the binding between ObgE and YbiB. The sample cell was filled with 75 μM ObgE while the syringe was loaded with 1 mM YbiB. The measurement was performed in triplicate. The resulting binding isotherms were fitted on a ‘two sets of sites’ model, with the ‘ligand in cell’ function activated, to determine affinities (*K*_D1_ and *K*_D2_) and stoichiometries (*n*_1_ and *n*_2_) for both binding events. Based on the three repeats, average *K*_D_-values (±SD) of respectively 22 ± 2 nM and 5 ± 2 μM are obtained. (**C**) Size exclusion chromatography (SEC) coupled to multi-angle light scattering (MALS) of ObgE (orange), YbiB (blue) and a YbiB-ObgE (1:3 molar ratio) mixture (green). The chromatograms display the scaled UV absorption at 280 nm (right y-axis), while the molar masses determined for the corresponding elution peaks (indicated in the same color) can be read from the left y-axis.

To confirm the interaction between YbiB and ObgE *in vitro*, first a pull-down experiment was performed. Purified C-terminally Twin-Strep-tagged ObgE (ObgE-Strep) protein was trapped on Strep-Tactin beads and used as bait, while N-terminally His-tagged YbiB protein was used as prey (Figure 2A and Supplementary Figure S3). This pull-down assay was performed for three different nucleotide states of ObgE-Strep (nucleotide free (NF), GDP-bound and GTPγS-bound). This experiment clearly shows that also *in vitro*, using the purified proteins, YbiB specifically interacts with ObgE, while no obvious effect of the nucleotide state of ObgE on this interaction is observed.

To investigate the interaction between ObgE and YbiB more quantitatively, ITC experiments were performed (in triplicate) by loading 75 μM N-terminally His_6_-tagged ObgE in the cell and 1 mM YbiB in the syringe (note: concentrations are always expressed as subunit concentrations, unless stated otherwise) (Figure 2B, Supplementary Table S6). Clear binding is observed, with the obtained binding isotherm displaying a biphasic interaction behavior, corresponding to a high-affinity (nanomolar) binding event and a low-affinity (low micromolar) binding event. When fitted on a ‘two sets of sites’ model, with the ‘ligand in cell’ function activated, average affinities (*K*_D_) of 22 ± 1 nM and 5 ± 1 μM are obtained for the first and second binding event, respectively. For each binding event, a stoichiometry (*n*) of around 0.5 is obtained, corresponding to one ObgE monomer binding to one of the YbiB subunits in the YbiB homodimer during each subsequent binding step. As a control, the YbiB dimer was titrated into buffer only (Supplementary Figure S4).

To confirm that two ObgE proteins indeed bind to one YbiB dimer as suggested by the ITC, we next performed size exclusion chromatography (SEC) coupled to multi-angle light scattering (MALS) (Figure 2C). To do so, a sample containing pre-mixed YbiB and ObgE in a 1:3 molar ratio was injected into the SEC column and compared to samples containing the individual proteins. The molar masses obtained through MALS for ObgE and YbiB are 47 (±0.2%) kDa and 75 (±0.1%) kDa, respectively, which is in very good agreement with the theoretical molar masses of an ObgE monomer (45 kDa) and a YbiB homodimer (75 kDa). Additionally, the homodimeric state of YbiB, even at very low concentrations, was confirmed using mass photometry (Supplementary Figure S5). For the YbiB-ObgE complex MALS yields a molar mass of 154 (±0.2%) kDa, just below the expected molar mass for a YbiB dimer bound to two ObgE proteins (166 kDa). This can be explained in light of our ITC experiments by a partial dissociation of the ‘low affinity’ ObgE molecule during gel filtration, yielding a mixture of YbiB-ObgE complexes with either one or two molecules of ObgE bound.

The results obtained through SEC-MALS were finally also confirmed through size exclusion chromatography coupled to small angle X-ray scattering (SEC-SAXS), using a pre-formed YbiB-ObgE complex (Supplementary Figure S6, Supplementary Table S4). Based on the Bayesian inference method (53), the SAXS data yield a molar mass interval for the complex between 152 and 177 kDa, corresponding again to the expected molar mass of two ObgE molecules bound to the YbiB dimer. Together, these ITC and SEC-MALS/SAXS results firmly establish that two ObgE monomers can bind to the YbiB homodimer, with one ObgE molecule binding with high affinity and the other with lower affinity.

### The ObgE C-terminal domain drives the interaction with YbiB

**Figure 3.**
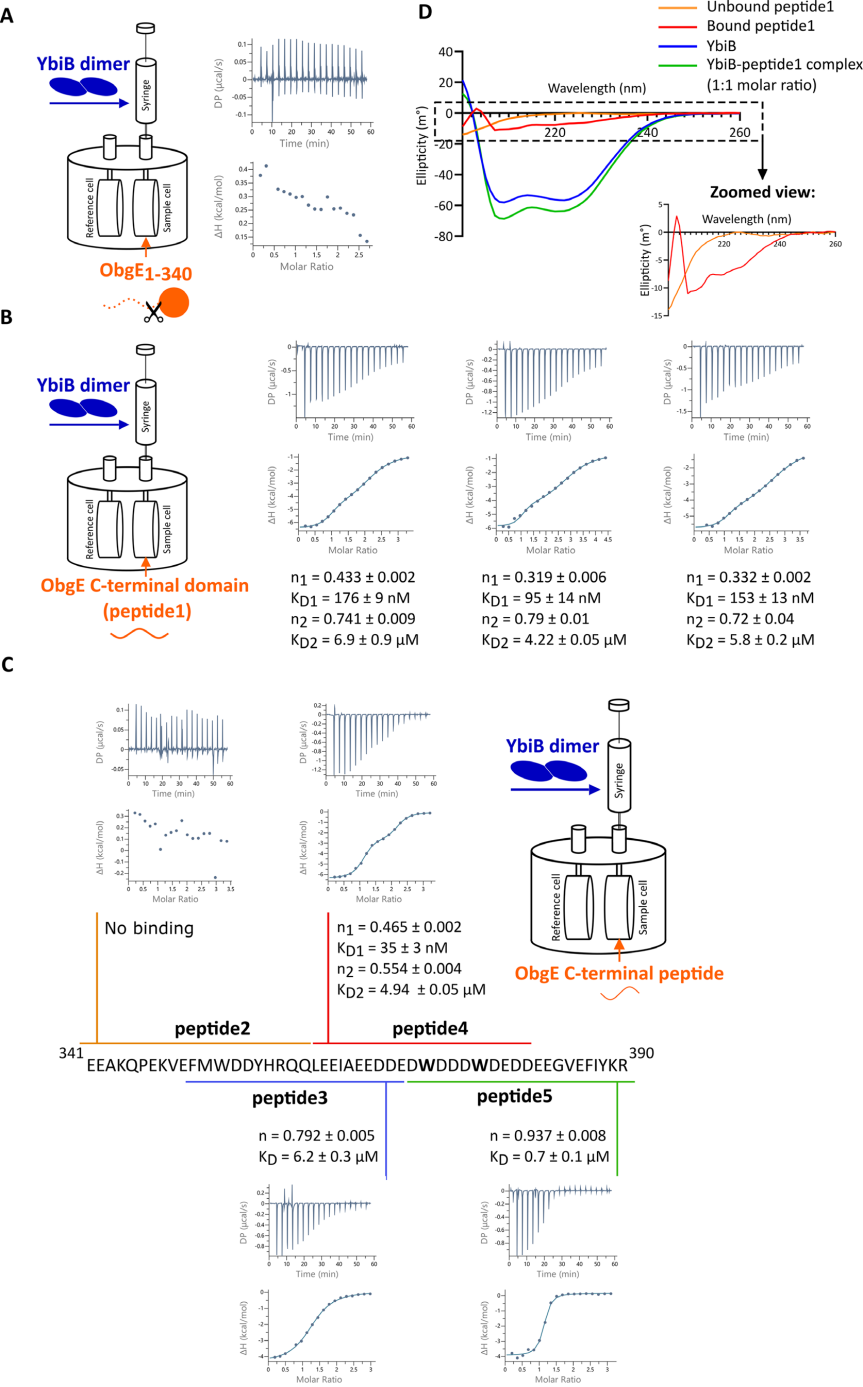
The ObgE C-terminal domain is important for the interaction with YbiB. (**A**) ITC measurement between the C-terminally truncated ObgE_1–340_ construct and YbiB. The sample cell was filled with 75 μM ObgE_1-340_ while the syringe was loaded with 1 mM YbiB. (**B**) ITC measurement between a synthesized peptide (peptide1), representing the entire intrinsically disordered C-terminal domain of ObgE, and YbiB. The sample cell was filled with 75 μM peptide1 while the syringe was loaded with 1.2–1.4 mM YbiB. The measurement was performed in triplicate. The resulting binding isotherms were fitted on a ‘two sets of sites’ model, with the ‘ligand in cell’ function activated, to determine affinities (*K*_D1_ and *K*_D2_) and stoichiometries (*n*_1_ and *n*_2_) for both binding events. Based on the three repeats, average *K*_D_-values (±SD) of respectively 141 ± 30 nM and 6 ± 1 μM are obtained. (**C**) ITC measurements performed between YbiB and different overlapping peptides of 20 amino acids that each cover a certain region of the intrinsically disordered C-terminal domain of ObgE. The sample cell was filled with 75 μM peptide while the syringe was loaded with around 1.3 mM YbiB. The resulting binding isotherms were fitted on a suitable model to determine affinities (*K*_D_) and stoichiometries (*n*). (**D**) CD spectra recorded for peptide1 (blue), YbiB (orange) and their complex (green). After subtracting the spectrum obtained for YbiB from the spectrum obtained for the complex, a CD spectrum for the bound C-terminal ObgE peptide is obtained (red). The inset shows a zoomed-in view of the CD spectra obtained for unbound (orange) and bound (red) peptide1.

The intrinsically disordered C-terminal domain of ObgE contains a high fraction of negatively charged residues (24 Asp or Glu residues out of 50 residues). Since the crystal structure of the YbiB dimer contains two strongly positively charged grooves on its surface (41), we reasoned that the C-terminal domain of ObgE could be important for the interaction with YbiB. To test this hypothesis, we used an ObgE construct lacking the last 50 amino acids (ObgE_1–340_) and tested binding to YbiB using ITC (Figure 3A). Since no binding signal is observed, we conclude that the C-terminal domain of ObgE is indeed crucial for the interaction with YbiB. Next, a peptide spanning the entire 50 amino acids of the ObgE C-terminus was synthesized (peptide1) (Supplementary Table S3), and binding to YbiB was again assessed using ITC. As is the case for full-length ObgE, a biphasic binding isotherm is observed for this peptide with *K*_D1_- and *K*_D2_-values of 141 ± 30 nM and 6 ± 1 μM, respectively (Figure 3B, Supplementary Table S6). While peptide1 clearly binds with high affinity to YbiB, the obtained K_D1_-value is increased 6-fold compared to the one obtained for full-length ObgE. This indicates that, while the C-terminal domain is the main driver of the interaction, also the presence of the other ObgE domains have an influence, either by participating directly in the interaction or by orienting the C-terminal domain.

To define the region within the ObgE C-terminal domain that is important for binding, several smaller peptides were synthesized (Supplementary Table S3), each representing overlapping windows of 20 amino acids of the ObgE C-terminus (Figure 3C). Using ITC, we find that peptide2, corresponding to the N-terminal 20 amino acid segment of the C-terminal domain, does not bind to YbiB. In contrast, the subsequent more C-terminally located peptides (peptide 3-5) do show binding (Figure 3C, Supplementary Table S6). Nevertheless, only peptide4 (corresponding to residues 361–380 of ObgE) displays the high-affinity biphasic binding behavior, establishing this region as a key epitope in the interaction with YbiB.

While the C-terminal domain of ObgE is overall highly polar and negatively charged, two bulky and hydrophobic tryptophan residues (Trp372 and Trp376) embedded within a cluster of Asp and Glu residues stand out (shown in bold in Figure 3C). Both Trp residues are present in peptide4. This immediately raises the question whether these Trp residues have a functional role and are important for the binding to YbiB. To verify this, variants of peptide4 (named peptide4-WW in this context) were synthesized in which either one Trp residue was substituted by Ala (peptide4-WA and peptide4-AW) or both (peptide4-AA). Removing both Trp residues clearly severely affects binding to YbiB since, in comparison to peptide4-WW, peptide4-AA completely loses the high affinity nanomolar binding step, and binds with an overall *K*_D_-value of about 10 μM (Supplementary Figure S7, Supplementary Table S6). Interestingly, both Trp residues seem to play a redundant role, since the substitution of only one of the two Trps maintains the biphasic high affinity binding and only has a minor impact on the affinity (Supplementary Figure S7, Supplementary Table S6).

Intrinsically disordered regions often fold upon binding to partner proteins (72). To verify whether the intrinsically disordered C-terminus of ObgE folds upon binding to YbiB, far-UV CD spectra were recorded for peptide1, YbiB and the YbiB-peptide1 complex (1:1 molar ratio) (Figure 3D). As expected, the CD spectrum of peptide1 shows a minimum around 200 nm, which is characteristic for a disordered peptide. On the other hand, the CD spectrum of YbiB displays the characteristics of a helical fold (minima around 208 and 222 nm), corresponding to its high α-helix content as shown by the crystal structure (41). The spectrum of peptide1 bound to YbiB can be approximated by subtracting the spectrum of YbiB from that of the YbiB-peptide1 complex. This resulting spectrum differs drastically from the spectrum obtained for the unbound peptide1 (inset of Figure 3D), showing minima around 207 and 219 nm, which indicates that the ObgE C-terminus becomes folded when bound to YbiB.

### Structural basis of the ObgE–YbiB interaction

**Figure 4.**
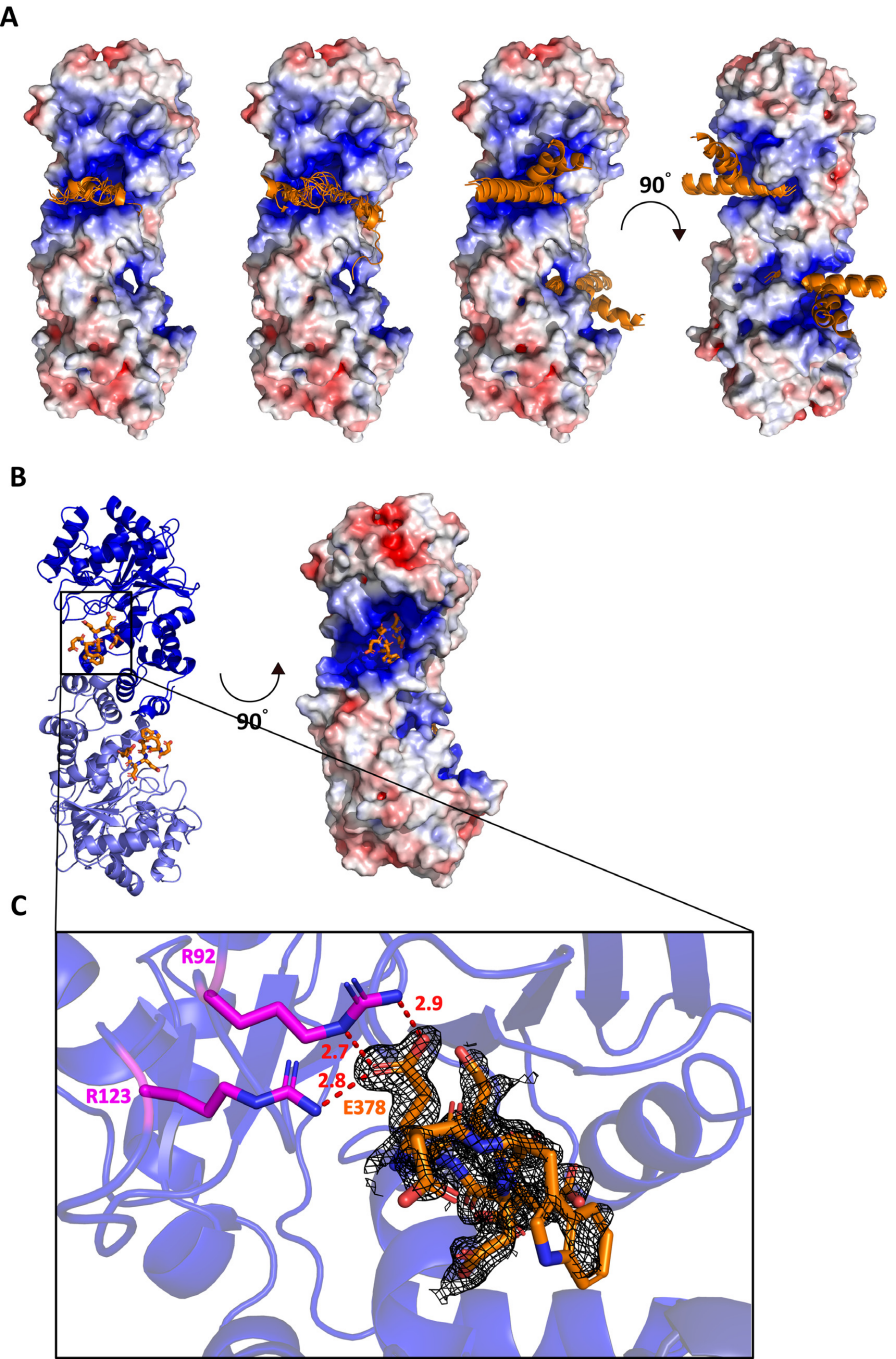
Structural basis of the interaction between YbiB and the ObgE C-terminal domain. (**A**) Docking models obtained for the YbiB dimer in complex with part of the ObgE C-terminus. *Left*: Top 10 docking models obtained by docking the ObgE C-terminal peptide ^370^EDWDDDWDE^378^ to the YbiB dimer using the motif-based ClusPro-PeptiDock program. *Middle*: Top 10 docking models obtained by docking the ObgE C-terminal peptide ^361^LEEIAEEDDEDWDDDWDEDDEE^382^ to the YbiB dimer using the CABS-dock program. *Right*: The 5 AlphaFold-Multimer models obtained for the YbiB dimer bound to the ObgE C-terminal peptide with sequence ^361^LEEIAEEDDEDWDDDWDEDDEE^382^. The electrostatic potential surface of the published YbiB structure (PDB: 4MUO) is superposed with the obtained models. Note: All docking solutions are shown on the same protomer within the YbiB dimer. (**B**) *Left*: Structure of the YbiB dimer in complex with part of the ObgE C-terminal peptide4 (^361^LEEIAEEDDEDWDD**DWDEDD**^380^, residues resolved in the structure are indicated in bold). One protomer of the YbiB dimer is colored blue, while the other protomer is colored in slate. The ObgE C-terminal peptide is shown as orange sticks. *Right*: Electrostatic potential surface representation of the YbiB dimer. Positively charged regions are colored blue, while negatively charged areas are colored red. The ObgE C-terminal peptide is shown as orange sticks. (**C**) Zoom-in on the ObgE C-terminal peptide (shown in orange sticks) displayed with its corresponding omit map contoured at 0.7σ (black mesh) bound to YbiB (blue cartoon). The Glu378 residue (E378, orange) seems to interact with Arg92 (R92, magenta sticks) and Arg123 (R123, magenta sticks) of YbiB. Possible interactions are represented by red dashed lines and the corresponding distances are displayed in Å.

So far, attempts to crystallize the complex of ObgE and YbiB remained unsuccessful. To gain insight in the molecular mechanism of the interaction between the C-terminal domain of ObgE and YbiB we therefore first resorted to molecular docking. Two peptide docking servers that use different approaches were used in parallel. On the one hand, the docking program ClusPro PeptiDock (56) was used to perform a motif-based docking of a short palindromic motif present in the ObgE C-terminus (^370^EDWDDDWDE^378^). On the other hand, CABS-dock was applied to dock a longer C-terminal ObgE peptide (^361^LEEIAEEDDEDWDDDWDEDDEE^382^), including the complete 17-residue-long palindrome of the ObgE C-terminus, to YbiB in an unbiased manner (59). As AlphaFold was recently shown to be a valuable alternative to docking programs for modeling protein-peptide interactions (73), AlphaFold-Multimer (54) was also used to generate models of YbiB bound to two copies of the latter peptide. Both docking strategies as well as the AlphaFold-Multimer modeling yield similar results, placing the peptide inside the positive grooves on YbiB’s surface (Figure 4A). This implies that, as expected, the negatively charged ObgE C-terminal domain indeed interacts with YbiB through its positively charged grooves. Moreover, as observed by the CD spectroscopy measurements, several docking poses, and especially the AlphaFold models, suggest that the peptide gains secondary structure upon binding to YbiB.

In addition to the docking, we also co-crystallized YbiB with C-terminal ObgE peptides for structure elucidation using X-ray crystallography. Co-crystallization of YbiB and peptide1 (spanning the complete C-terminal domain of ObgE) only yielded crystals that contained YbiB in absence of the peptide. Nevertheless, this allowed us to solve the structure of apo YbiB to a resolution of about 1.3 Å, which is higher than the currently available YbiB structure (Supplementary Table S5 and Supplementary Figure S8) (41). Similar to the previously published YbiB structure (PDB: 4MUO (41)) YbiB forms a head-to-head dimer, while the current high-resolution structure also resolves a previously missing loop (comprising residues 258–267) (Supplementary Figure S8). Subsequently, we successfully obtained crystals in complex with the shorter peptide4, which resulted in a dataset and electron density map at near-atomic resolution (Supplementary Table S5). In both YbiB subunits clear electron density is present in the expected binding site of the peptide; however, this density only accounts for a part of peptide4, which was interpreted as amino acid residues ^375^DWDEDD^380^. This indicates that the rest of the peptide might be more flexible or that alternative ways of binding to YbiB are possible for those regions. In agreement with the docking results, the peptides are bound in the positively charged grooves close to the dimer interface of YbiB (Figure 4B). Within the visible peptide stretch, the glutamate residue corresponding to Glu378 is resolved the best, and the structure shows close interaction of this glutamate with residues Arg92 and Arg123 of YbiB (Figure 4C). Interestingly, both arginine residues are highly conserved among the TrpD2 family according to the ConSurf web server (Supplementary Figure S9) (67,68,74–76).

The involvement of both arginine residues in the interaction with ObgE was further validated via site-directed mutagenesis. Both residues were mutated to glutamate and binding to ObgE was measured using ITC (Supplementary Figure S10). In agreement with an important role in ObgE binding, the Arg92Glu and Arg123Glu mutants lose the high-affinity biphasic binding to ObgE and average *K*_D_ values (± SD) of 10.7 ± 0.6 and 6 ± 3 μM are found, respectively.

### YbiB does not display GAP activity towards ObgE

**Figure 5.**
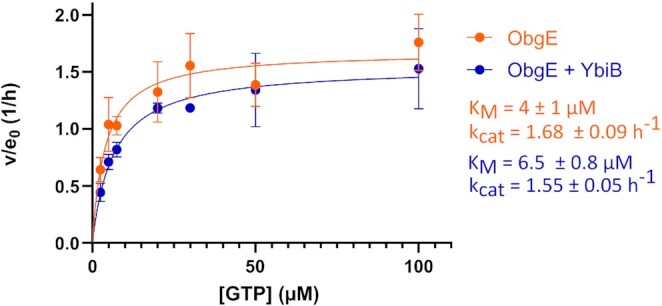
YbiB does not function as a GAP of ObgE. Steady-state initial rate kinetics (Michaelis–Menten) data obtained for GTP hydrolysis by ObgE in absence (orange) and presence (blue) of YbiB. Measurements were performed in triplicate. Steady-state kinetics parameters, obtained by fitting the curves on the Michaelis-Menten equation, are displayed ± SEM.

G-proteins, such as ObgE, are commonly regulated via GAP and GEF proteins that affect their GTPase activity and nucleotide exchange, respectively (1,4,5). While ObgE displays an intrinsically fast nucleotide exchange, probably making GEFs obsolete, its intrinsic GTP hydrolysis is slow (7). Nevertheless, with the exception of the ribosome that stimulates ObgE GTP hydrolysis (7,11), no GAPs are currently described for ObgE. Since we establish YbiB as a novel ObgE interactor, we next tested whether binding of YbiB influences ObgE GTP hydrolysis. Steady-state kinetic assays were performed by incubating ObgE with different concentrations of GTP in absence and presence of a 1:10 molar excess of YbiB. The amount of GDP formed through GTP hydrolysis by ObgE was determined at different time points of the reaction using a reversed-phase chromatography setup (7). When comparing ObgE’s activity in absence and presence of YbiB, we can conclude that YbiB does not significantly alter the steady-state kinetic parameters, *k*_cat_ and *K*_M_, of GTP hydrolysis by ObgE, and hence does not function as a GAP (Figure 5).

### ObgE inhibits DNA binding to YbiB

**Figure 6.**
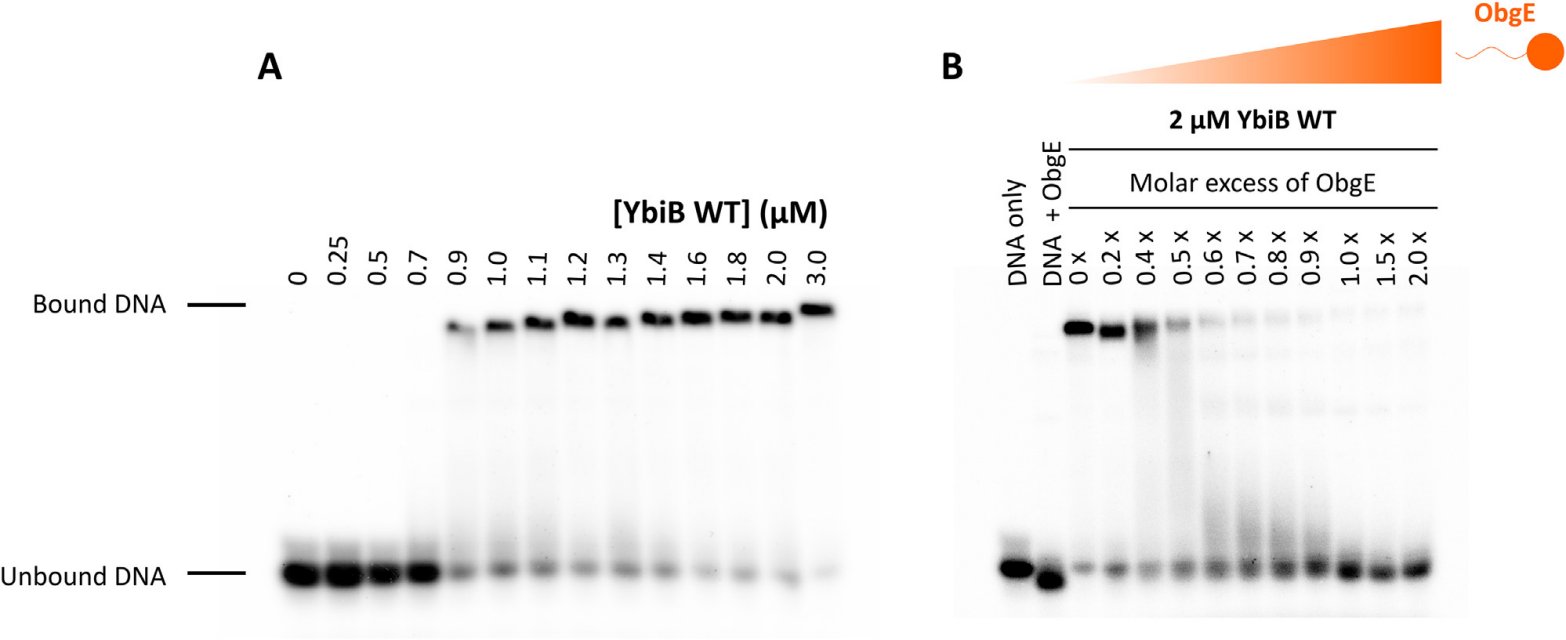
ObgE inhibits DNA binding to YbiB. Electrophoretic mobility shift assays (EMSAs) performed for YbiB WT in absence (**A**) and presence (**B**) of ObgE. (**A**) A ^32^P-labeled ssDNA strand of 58 base pairs was incubated with increasing concentrations of YbiB. (**B**) The same ^32^P-labeled ssDNA probe was incubated with 2 μM of YbiB and increasing concentrations of ObgE. As a control, the DNA probe was also incubated with the highest concentration of ObgE in absence of YbiB. All samples were analyzed on a 6% polyacrylamide gel.

It has previously been shown that YbiB binds both single- and double-stranded DNA in a seemingly sequence-independent manner and with comparable affinities (41). Although the exact DNA-binding groove on YbiB has not yet been established, it is likely that the negatively charged phosphate backbone of the DNA interacts with the positively charged surface of the YbiB dimer. Since we show that the C-terminal domain of ObgE also binds in this positively charged pocket, we next tested whether ObgE interferes with YbiB DNA binding. First, DNA binding to YbiB was confirmed with an electrophoretic mobility shift assay using a ^32^P-labeled ssDNA probe with a sequence identical to one of the YbiB-binding probes that was previously reported (41) (Figure 6A and Supplementary Figure S11). A clear shift of the DNA band is indeed observed upon mixing with increasing amounts of wild-type YbiB and fitting of the data yields an apparent *K*_D_ of approximately 0.85 μM, which is 3–4 times higher than the *K*_D_-value previously reported (41). Next, a fixed concentration of YbiB at which DNA binding was nearly saturated (2 μM) was pre-mixed with increasing concentrations of ObgE and DNA binding was assessed using the same assay. This EMSA shows that ObgE very efficiently inhibits DNA binding to YbiB (Figure 6B). Intriguingly, nearly complete inhibition of DNA binding is already observed at a 1:2 ObgE/YbiB molar ratio (expressed as subunit concentrations), suggesting that one ObgE molecule blocks binding of the DNA probe to both DNA binding pockets on the YbiB dimer.

To further confirm that YbiB uses the same pocket to bind to DNA and ObgE, DNA binding to the YbiB Arg92Glu and Arg123Glu mutants was tested. As we previously demonstrated, both residues directly interact with ObgE. EMSA shows that both mutations also severely affect YbiB’s DNA-binding capacity with apparent *K*_D_-values that are >10 times higher than the one of wild-type YbiB (Supplementary Figure S11). This thus confirms that, at least partially, the same YbiB residues are used to bind DNA and ObgE.

## DISCUSSION

Members of the Obg subfamily of G-proteins, and the *E. coli* representative ObgE in particular, function at the crossroads of a large plethora of cellular functions, including ribosome assembly/maturation, DNA replication, cell division, stress response and persistence (6,10). Based on this wide variety of functional roles one would expect that ObgE acts as a cellular hub that interacts with a large set of regulatory proteins and downstream effectors. Nevertheless, aside from several ribosomal proteins, only a relatively limited number of ObgE interactors has been described and characterized in literature so far (16,17). Here, we identified and subsequently characterized the DNA-binding TrpD2 protein YbiB as a novel interactor of ObgE. After the initial identification using *in vivo* crosslinking, the interaction between the two proteins was further confirmed through bacterial two-hybrid and pull-down assays. Subsequently, the ObgE–YbiB interaction was characterized in detail using biochemical, computational and structural biology approaches.

SEC-MALS and SEC-SAXS analyses show that YbiB and ObgE form a 2:2 complex, with two ObgE molecules binding to one YbiB homodimer. However, intriguingly, ITC measurements demonstrate that YbiB and ObgE interact in a characteristic biphasic manner, with affinities in the low nanomolar range for one binding event and in the low micromolar range for the other binding event (Figure 2). A priori, different theoretical scenarios could be envisioned to explain such a biphasic interaction behavior (Supplementary Figure S12). In a first scenario, the ObgE binding sites present on the YbiB homodimer would partially overlap, causing the binding of one ObgE molecule to sterically hinder the binding of a second ObgE molecule. A similar type of behavior was previously observed for binding of the antitoxin-derived peptide CcdA^37–72^ to the homodimeric CcdB toxin (77). Here, the structure of the CcdA^37–72^-CcdB complex showed that binding of a first CcdA^37–72^ peptide on CcdB precludes the binding of the C-terminal part (amino acids 64–72) of the second peptide due to an overlap between the binding sites. This results in the second peptide binding with lower affinity, explaining the observed biphasic ITC binding isotherms. On the other hand, the biphasic binding behavior between ObgE and YbiB could also be explained by an allosteric mechanism in which binding of a first ObgE molecule to the YbiB dimer causes a conformational change in the second subunit toward a lower affinity state. Interestingly, and in line with such an allosteric mechanism, YbiB has previously been suggested to display negative cooperativity with regards to DNA binding, seemingly binding DNA with only one of the two DNA binding sites on the dimer (41). As a final formal possibility, the YbiB dimer might also harbor two pre-existing different binding sites for ObgE. However, we deem the latter as unlikely since the crystal structure shows YbiB as a completely symmetrical homodimer (PDB: 8BFR).

To gain more insight into the mechanism underlying the observed biphasic behavior, allowing to discriminate between the alternative scenarios, we investigated the YbiB-ObgE interaction in more detail. A particular region within the negatively charged and intrinsically disordered C-terminus of ObgE, comprising a palindromic amino acid sequence, was identified as the main driver of the interaction with YbiB (Figure 3). In agreement with docking studies, a crystal structure of YbiB in complex with a peptide spanning part of this ObgE C-terminal region shows that the C-terminal domains of ObgE bind at the interface of the YbiB dimer in the positively charged and partially interconnected grooves on YbiB’s surface (Figure 4). This location of the ObgE binding sites strongly favors the scenario of overlapping binding sites to explain the biphasic binding behavior between YbiB and ObgE, in analogy with the CcdA/B interaction mode. Moreover, superposition of the YbiB-peptide4 and YbiB-apo structures does not reveal any significant structural changes within the YbiB dimer upon binding of peptide4, arguing against an allosteric mechanism. A particular feature of the ObgE intrinsically disordered C-terminal domain is the presence of two tryptophan residues amidst a stretch of glutamates and aspartates. ITC experiments show that the tryptophan residues are crucial for the high-affinity binding of ObgE to YbiB, while the presence of only one of both tryptophans seems sufficient to maintain this high-affinity interaction. We hypothesize that this might be due to the palindromic sequence surrounding the tryptophan residues (^366^EEDDEDWDDDWDEDDEE^382^), possibly allowing the C-terminus of ObgE to bind within the positive clefts on the YbiB surface in different, slightly altered—either shifted or inverted—ways. This would also explain the rather weak electron density that is observed for the C-terminal ObgE peptide in the YbiB-peptide crystal structure. Nevertheless, the latter crystal structure also allowed us to identify two arginine residues (Arg92 and Arg123) of YbiB that are important for the high-affinity interaction with ObgE. These arginine residues are strongly conserved within the TrpD2 family according to the ConSurf Web Server (67), suggesting that they play an important functional role.

While these results firmly establish YbiB as a new ObgE interaction partner, the exact functional implications and roles of this interaction—and of YbiB as such—require more investigation. In general, GTPases are commonly regulated by GAP and GEF proteins, while for ObgE regulation by GEFs is most probably obsolete due to its intrinsic low nucleotide affinity and fast nucleotide exchange, similar to many other bacterial GTPases (78,79). In this study we excluded a role of YbiB as a GAP protein for ObgE (Figure 5), leaving (to the best of our knowledge) the ribosome as the only currently described GAP protein for ObgE (7,11). A clear functional link between ObgE and YbiB is however revealed by our EMSA experiments to study YbiB DNA binding (Figure 6). It was established before that YbiB binds DNA in a seemingly sequence-independent way (41), and we show that the YbiB residues Arg92 and Arg123 play a common role in DNA and ObgE binding, suggesting a shared ObgE/DNA binding pocket. Correspondingly, we find that ObgE very potently competes with DNA-binding to YbiB. Strong inhibition of DNA binding by YbiB already occurs at a 1:2 ObgE/YbiB molar ratio (expressed as subunit concentrations), implying that one ObgE molecule can block binding of the DNA probe to both DNA-binding pockets on the YbiB dimer. This observation further suggests that both positively charged clefts of the YbiB homodimer contribute to the ObgE binding site, which again favors the model of partially overlapping ObgE binding sites to explain the biphasic interaction behavior (Supplementary Figure S12).

A final and very intriguing functional link between YbiB and ObgE, is provided by our finding that overexpression of *ybiB* leads to an increased persister fraction, similar to, but less pronounced than, ObgE (Figure 1). This persistence-related effect of YbiB seems, however, not to be situated directly downstream or upstream of ObgE, since ObgE is still able to induce persistence in a *ybiB* knock-out strain. Considering that the *E. coli ybiB* gene is part of an operon that is under control of the LexA repressor and is induced upon DNA damage (41), it is probable that YbiB plays a so far unknown role in the SOS stress response. YbiB may thus form a link between ObgE and the SOS response, which has been observed before (30–32). However, it is also still formally possible that YbiB solely exploits the expression level of the LexA-regulated operon while serving another cellular function, according to the ‘genomic hitchhiking’ phenomenon (41,80). Further studies to reveal the cellular function of YbiB will be necessary to fully comprehend the physiological implications of the YbiB–ObgE interaction. Nevertheless, the current findings provide a new important piece of the puzzle toward understanding the complex regulatory cellular role of ObgE.

## DATA AVAILABILITY

Source datasets are deposited on the online repository Zenodo with identifier 10.5281/zenodo.7614560. Atomic coordinates and structure factors for the reported crystal structures have been deposited with the Protein Data Bank under accession codes 8BFR and 8BFT. Small angle X-ray scattering data were deposited to the Small Angle Scattering Biological Data Bank (SASDB) under accession code SASDQ78.

## Supplementary Material

gkad127_Supplemental_File
