# Supplementary material for: YbiB: a novel interactor of the GTPase ObgE
Source: Nucleic Acids Res. 2023 Mar 2;51(7):3420–35. doi: 10.1093/nar/gkad127 (PMC10123104; doi:10.1093/nar/gkad127)
Supplement: gkad127_Supplemental_File [file gkad127_supplemental_file.pdf]

Supplementary Information

YbiB: a Novel Interactor of the GTPase ObgE

Babette Deckers, Silke Vercauteren, Veerke De Kock, Charlotte Martin, Tamas Lazar, Pauline Herpels, Liselot Dewachter, Natalie Verstraeten, Eveline Peeters, Steven Ballet, Jan Michiels, Christian Galicia, Wim Versées

SUPPLEMENTARY METHODS

Identification of ObgE interaction partners by photoreactive crosslinking

Sample preparation

The construction of pBAD/His A-*obgE* and pBAD/His A-*obgE*$_{D246G}$ has previously been described (1,2). Amber codons were introduced into pBAD/His A-*obgE* and pBAD/His A-*obgE*$_{D246G}$ using the QuikChange Site-Directed Mutagenesis Kit (Stratagene) and checked by Sanger sequencing. The pSup-BpaRS-6TNR(D286R) plasmid encoding the suppressor tRNA and an engineered aminoacyl-tRNA synthetase charging the suppressor tRNA with *p*Bpa (3) was a gift from Peter Schultz (the Scripps Research Institute). Plasmids containing an amber codon artificially introduced in *obgE* or *obgE*$_{D246G}$ were transformed into chemocompetent DH10B cells containing the pSup-BpaRS-6TNR(D286R) plasmid. Cultures were grown overnight at 37°C in Lysogeny Broth (LB) supplemented with 100 µg/ml ampicillin and 15 µg/ml chloramphenicol while shaking at 200 rotations per minute (rpm). Subsequently, the overnight cultures were diluted 1:100 in 500 ml LB supplemented with 100 µg/ml ampicillin and 15 µg/ml chloramphenicol and incubated at 37°C until the OD (595 nm) reached 0.5. Crosslinker *p*Bpa (1 mM, H-p-Bz-Phe-OH F-2800.0005 Bachem AG) was added from a 1 M stock solution dissolved in 1 M NaOH and incubated for 30 min at 37°C and 200 rpm. Expression of *obgE* alleles was induced with 0.2% arabinose for 4 h at 37°C and 200 rpm. The cells were collected by centrifugation for 20 min at 4500 g after which the supernatant was discarded. The pellet was suspended in 5 ml 1x phosphate buffered saline (PBS; 137 mM NaCl, 10 mM phosphate, 2.7 mM KCl, pH 7.4) and the suspension was transferred to a Petri dish. The samples were irradiated with UV light (ReproSet™, Amersham Biosiences) at 5 cm distance from the light source for 40 min in timeframes of 10 min with intermitted mixing. The suspension was placed close to the UV lamp for optimal activation of the crosslinker. Next, the samples were transferred to a falcon tube and centrifuged for 15 min at 4500 g to pellet the cells. The cell pellet was stored at -20°C to stimulate cell lysis.

EDTA-free protease inhibitor cocktail complex (cOmplete™, Merck) dissolved in 1x PBS was added to the pellet in order to protect the proteins during sample preparation. For each gram of cell pellet, 250 units of benzonase (Merck) were added. The sample underwent sonication followed by low-speed centrifugation (4500 g for 10 min at 4°C) to remove cell debris. High-speed centrifugation (250000 g for 20 min at 4°C) was used to separate the soluble and insoluble protein fraction. The insoluble fraction was suspended in 2 ml 8 M GnHCl. Several sonication bursts were used until the suspension was completely solubilized.

Purification of ObgE interaction complexes

For each sample, 100 µl of Co^{2+} IDA agarose resin (HisPur™ Cobalt Resin, ThermoFisher Scientific™) was equilibrated in wash buffer A (50 mM Tris, 1 M NaCl, 5% glycerol, 10 mM imidazole, 1 mM β-mercaptoethanol, 6 M urea, pH 7.4). The lysed protein sample containing His_6-tagged ObgE was added to the resin beads and incubated rotating for 30 min at 16°C. Next, the sample and resin beads mixture was loaded on a spin column (Pierce™ Spin Columns, ThermoFisher Scientific™). To remove excess proteins, the beads were washed two times with wash buffer A (10x resin volume), followed by two wash steps (10x resin volume) with wash buffer B (50 mM Tris, 50 mM NaCl, 5% glycerol, 10 mM imidazole, 1 mM β-mercaptoethanol, 6 M urea, pH 7.4). The protein complexes were eluted by competition with imidazole in five consecutive elution steps with 100 µl of buffer C (50 mM Tris, 50 mM NaCl, 5% glycerol, 200 mM imidazole, 1 mM β-mercaptoethanol, 6 M urea, pH 7.4), after a 30 min incubation period per step. The samples were stored at -20°C until further analysis.

Visualization of ObgE interaction complexes

11 µl of protein sample was mixed with NuPAGE LDS Sample Buffer and Reducing Agent, incubated at 70°C for 10 min and loaded onto a NuPAGE Novex Bis-Tris Mini gel (Invitrogen). Gels were run in MOPS running buffer (50 mM MOPS, 50 mM Tris, 1 mM EDTA, 0.1% sodium dodecyl sulfate (SDS) (w/v), pH 7.7) at 200 V for 35 min. The gels were stained using a Mass spectrometry (MS)-compatible silver staining. For this purpose, the gel was incubated in Fix solution (100 ml CH_3OH, 10 ml CH_3COOH, 90 ml Milli-Q water) for 20 min and subsequently incubated in Wash solution (100 ml CH_3OH, 100 ml Milli-Q water) for 10 min and rinsed with 200 ml Milli-Q water for 10 min. The gel was sensitized in sensitizing solution (0.04 g $Na_2S_2O_3$, 200 ml Milli-Q water) for 1 min. The gel was rinsed with 200 ml Milli-Q water for 2 x 1 min, followed by staining with pre-chilled silver nitrate solution (0.2% $AgNO_3$, 200 ml Milli-Q water) for 20 min at 4°C. The gel was rinsed again and incubated with developer solution (0.08 ml 10% Formalin, 4 g Na_2CO_3, 200 ml Milli-Q water) by intensive shaking. When the solution turned yellow, it was removed and fresh developer was added. The development was stopped by adding 200 ml of 5% CH_3COOH. Subsequently, the gel was stored in 200 ml of 1% CH_3COOH at 4°C (4). Moreover, in parallel, duplicates of the gels were stained in SilverBlue Staining to check for impurities. Gels were incubated in staining solution (0.12% Coomassie G-250, 10% $(NH_4)_2SO_4$, 10% H_3PO_4 and 20% CH_3OH) for one hour on a shaking platform at room temperature. Subsequently, the gels were washed in destain solution (10% $(NH_4)_2SO_4$, 10% H_3PO_4 and 20% CH_3OH) until bands became visible and the background signal was removed (5).

Finally, to confirm the production of full-length ObgE proteins, western blots were performed using monoclonal anti-His_6 antibodies (Roche) and Anti-Mouse IgG Alkaline Phosphatase-Conjugated antibodies (Sigma). Proteins on the gel were transferred to a polyvinylidene fluoride (PVDF) membrane using preassembled Trans-Blot Turbo Transfer Packs and a Trans-Blot Turbo Transfer System (Bio-Rad). The voltage was set to 15 V and samples were run for 3 min. After transfer, the membrane was incubated for one hour in the presence of blocking solution (1 g BSA in 100 ml Tris-buffered saline (TBS) buffer: 150 mM NaCl, 50 mM Tris, pH 7.5). The membrane was washed 3 times in 50 ml TBS for 10 min, after which monoclonal anti-His_6 antibodies (Roche) diluted in blocking solution were added. Following overnight incubation (50 rpm, 4°C), the membrane was washed 3 times for 5 min with 50 ml TBS containing 0.1% Tween 20 (v/v) and incubated for one hour (50 rpm) in the presence of Anti-Mouse IgG Alkaline Phosphatase-Conjugated antibodies (Sigma) diluted in blocking solution. The membrane was again washed 3 times for 10 min with 50 ml TBS containing 0.1% Tween 20 and finally incubated in substrate solution (5 mg NBT and 2.5 mg BCIP dissolved in 15 ml AP buffer: 0.1 M NaCl, 0.1 M Tris, pH 9.5) (without shaking). The reaction was stopped by adding 100 ml 20 mM EDTA in PBS. Unless indicated otherwise, all incubations were performed at room temperature, while shaking at 100 rpm.

In-gel trypsin digestion

Protein bands of interest were excised from the MS-compatible silver-stained gel and cut into small pieces. The gel was washed twice with Milli-Q water while vigorously shaking. Next, the mixture was briefly centrifuged for 10 s at 16000 g and the supernatant (containing excess SDS) was carefully discarded. The gel was submitted to a second wash step with 100 mM ammonium bicarbonate to give the gel a basic pH, required for alkylation. Subsequently, 10 mM Tris(2-carboxyethyl)phosphine (TCEP) was added until the gel slices were completely covered. The mixture was incubated at 62°C for 30 min before centrifugation (10 s at 16000 g) and the supernatant was discarded. TCEP reduces disulfide bonds, which is essential for subsequent cysteine alkylation. Immediately after, 55 mM iodoacetamide (IAA) was added and samples were incubated for 30 min in the dark at room temperature, gently tumbling. After centrifugation (10 s at 16000 g), the supernatant was discarded. The gel slices were washed three times with 50:50 acetonitrile (ACN): 100 mM ammonium bicarbonate while vigorously shaking, after which the supernatant was discarded again following another round of centrifugation

(10 s, 16000 g). 100% ACN was added to the dry gel slices, after which ACN was removed by a vacuum concentrator (SpeedVac). Trypsin solution (lyophilized trypsin in trypsin resuspension buffer, 10 ng/µl) was added to the gel slices and incubated for 10 min at room temperature. The slices were then covered with 25 mM ammonium bicarbonate and incubated overnight under constant shaking at 37°C. The samples were briefly centrifuged (10 s at 16000 g) and the supernatant was transferred to a new Eppendorf tube. In four subsequent steps, 5% formic acid was added to the remaining gel slices. Following 15 min incubation at room temperature, the supernatant was removed and combined with the initial supernatant sample. C18 Stage Tips (Proxeon, Thermo Scientific) were placed in the hole of the lid of a screw capped 2 ml Eppendorf tube. The Stage Tips were conditioned with 50 µl MeOH to improve protein binding to the membrane. Then, 50 µl of STSB wash buffer (0.5% (vol/vol) formic acid in 80% (vol/vol) ACN/Milli-Q water) was added followed by centrifugation (1 min, 800 g). This step was subsequently repeated with STSA wash buffer (0.5% (vol/vol) formic acid in Milli-Q water). 90 µl of STSA was added per 10 µl of MS sample. The samples were loaded on the Stage tip and centrifuged for 2 min at 800 g. The flow-through was re-applied to the tip and centrifuged a second time. The StageTip now contained immobilized peptides and was washed with 100 µl STSA before elution with 50 µl STSB. The resulting eluate was placed under the SpeedVac to remove excess ACN and kept at 4°C for immediate MS analysis.

Liquid chromatography coupled with tandem mass spectrometry

The first two elution fractions of every sample were combined and sent out for shotgun liquid chromatography coupled with tandem mass spectrometry (LC-MS/MS) at the VIB Proteomics Core (Ghent, Belgium). Samples were run in triplicate. Purified proteins underwent in-house proteolytic digestion and desalting. The desalted peptides were injected into the LC-MS/MS system for shotgun analysis, which consists of peptide identification and protein inference. For peptide identification, the experimental tandem mass spectra was searched using the MaxQuant algorithm (version 1.5.8.3) (6) with mainly default search settings including a false discovery rate set at 1% on both the peptide and protein level. Spectra were searched against the *E. coli* K-12 protein sequences in the Swiss-Prot database (database release version of January 2018 containing 4313 *E. coli* protein sequences) (www.uniprot.org).

The identified peptides were assembled into a set of confident proteins. In all 60 samples (ObgE, $ObgE_{W122}$, $ObgE_{I250}$, $ObgE_{Y269}$, $ObgE_{Y388}$, $ObgE_{D246G}$, $ObgE_{D246G,W122}$, $ObgE_{D246G,I250}$, $ObgE_{D246G,Y269}$, $ObgE_{D246G,Y388}$; with and without *p*Bpa; three replicates each), a total of 853631 peptides were identified. To compare protein intensities in the sample with and without *p*Bpa, a t-test was performed (FDR = 0.05 and S_0 = 1). Proteins that were significantly upregulated in ObgE samples that were supplemented with *p*Bpa compared to proteins detected in the ObgE samples who received no *p*Bpa were listed per construct. This way, over 400 interactors were selected as putative ObgE-interactors. Subsequently, the same approach was followed for the $ObgE_{D246G}$ samples. Then, for each construct, only the proteins interacting with ObgE and not with $ObgE_{D246G}$ were retained for further analysis. Also proteins interacting with the negative control ObgE or $ObgE_{D246G}$ (without an amber codon substitution) were removed.

SUPPLEMENTARY FIGURES

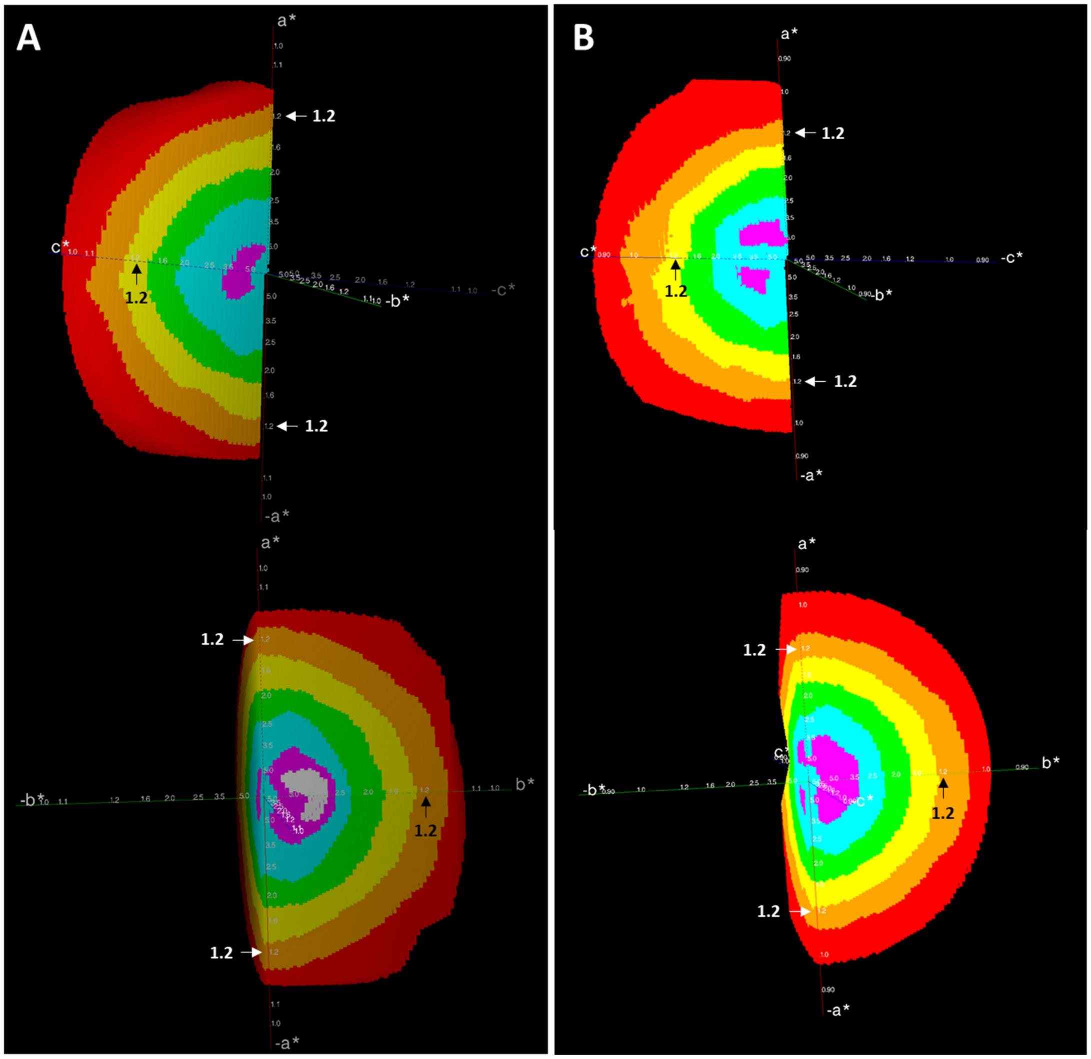

Figure S1 Reciprocal lattices (axes a*, b*, c*) color coded by mean I/σ(I) as given by STARANISO, showing the anisotropic diffraction of the crystal of (A) apo YbiB and (B) YbiB bound to ObgE *C*-terminal peptide4. For the YbiB-apo structure, diffraction limits of 1.45 Å along a*-0.027c*, 1.36 Å along b* and 1.27 Å along -0.140a*+0.990c* were obtained. For the YbiB-peptide4 structure, diffraction limits of 1.44 Å along a*+0.004c*, 1.33 Å along b* and 1.19 Å along -0.363a*+0.932c* were obtained. This anisotropy was taken into account in the further processing of both datasets. For clarity, the 1.2 Å resolution limit is indicated on the reciprocal axes.

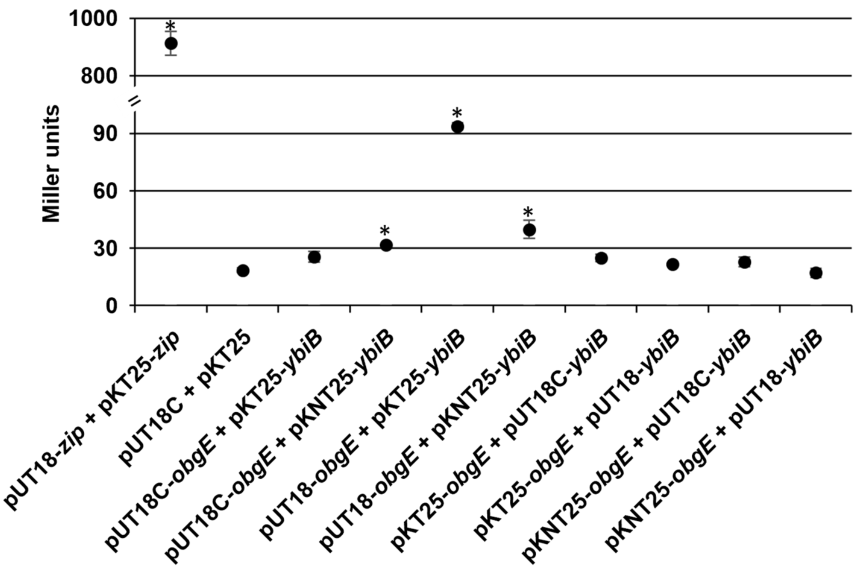

Figure S2 B2H assay to validate the ObgE-YbiB interaction. A strain harboring pUT18-*zip* and pKT25-*zip* served as a positive control, while a strain harboring pUT18C and pKT25 was used as a negative control. Combinations generating Miller Units that significantly differ from the negative control (pUT18C + pKT25) are indicated by an * ($p < 0.01$). Means ± SEM are presented ($n \geq 3$).

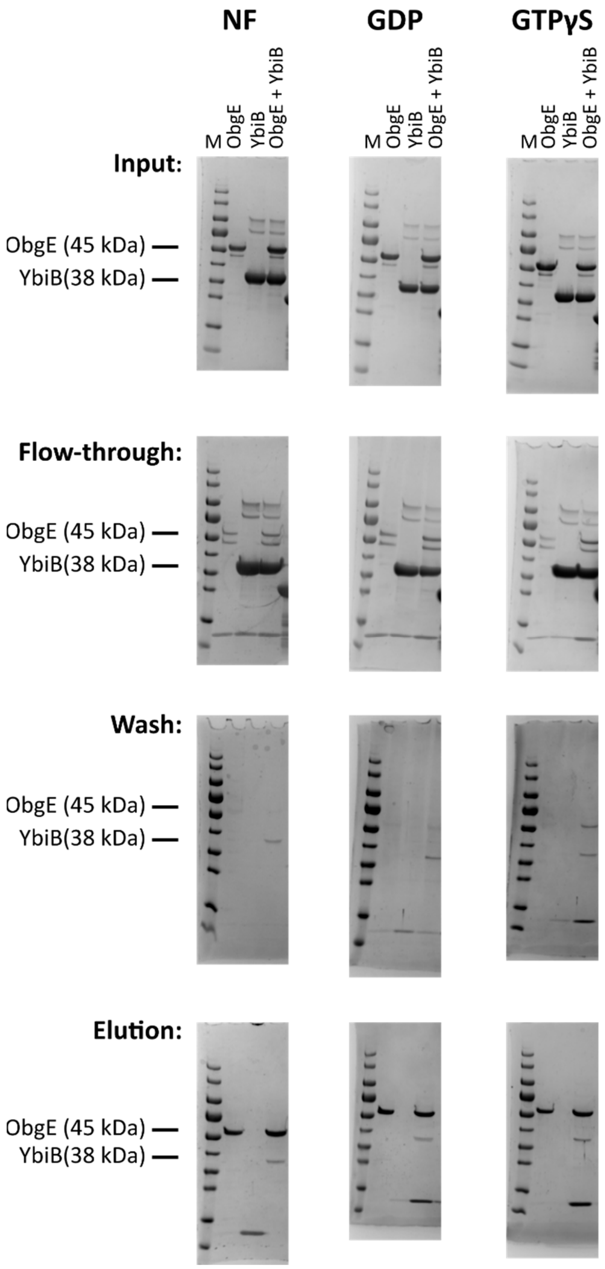

Figure S3 Complete overview of SDS-PAGE gels displaying the result of the different steps performed during the *in vitro* pull-down assays shown in Fig. 2A. C-terminally Strep-tagged ObgE protein was trapped on Strep-Tactin beads and used as bait, while N-terminally His_6-tagged YbiB protein was used as prey. As a control, the Strep-Tactin beads were also incubated with only ObgE or YbiB. The flow-through was collected and, after performing 6 wash steps, the proteins were eluted with buffer containing desthiobiotin. The experiment was performed for different nucleotide states of ObgE (nucleotide free (NF), GDP- and GTPγS-bound).

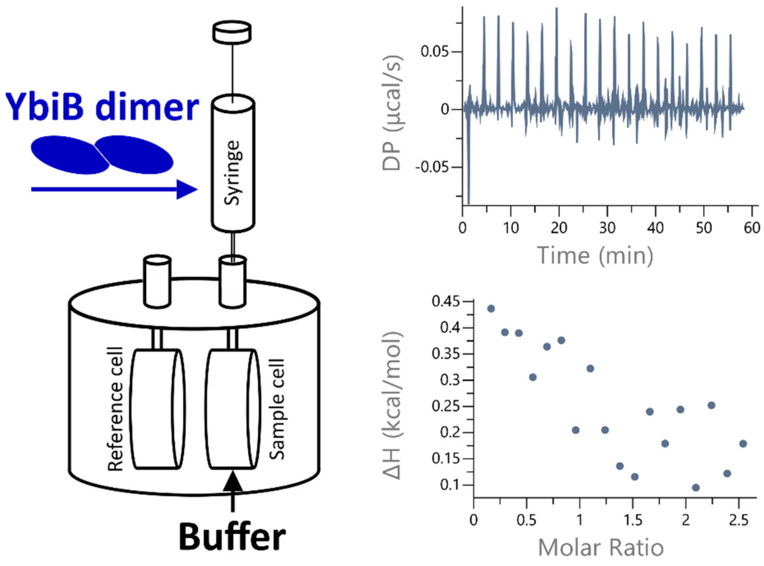

Figure S4 Titration of 1 mM YbiB into buffer, used as a control experiment

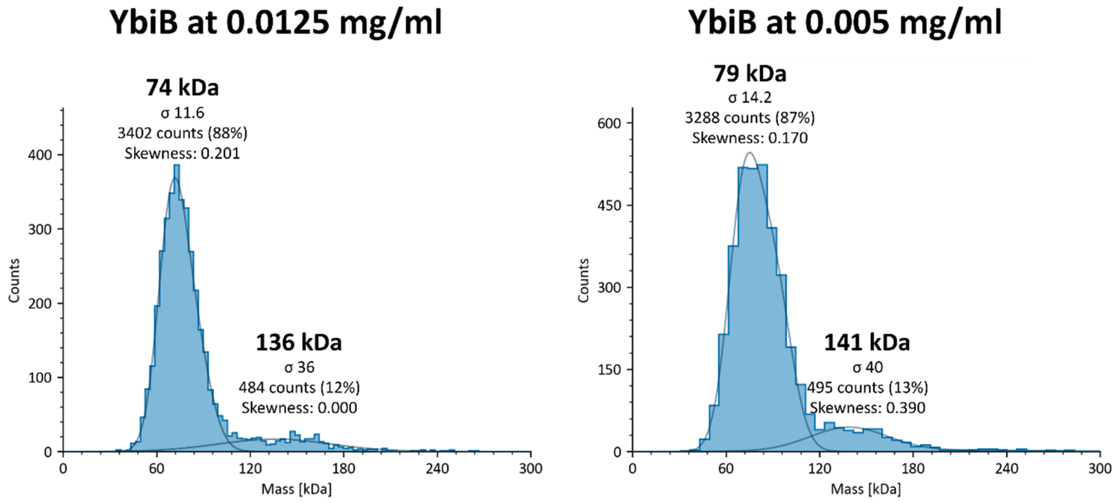

Figure S5 Mass photometry measurements to assess the molecular mass of YbiB at low concentration (0.0125 mg/ml and 0.005 mg/ml), confirming a stable YbiB homodimer.

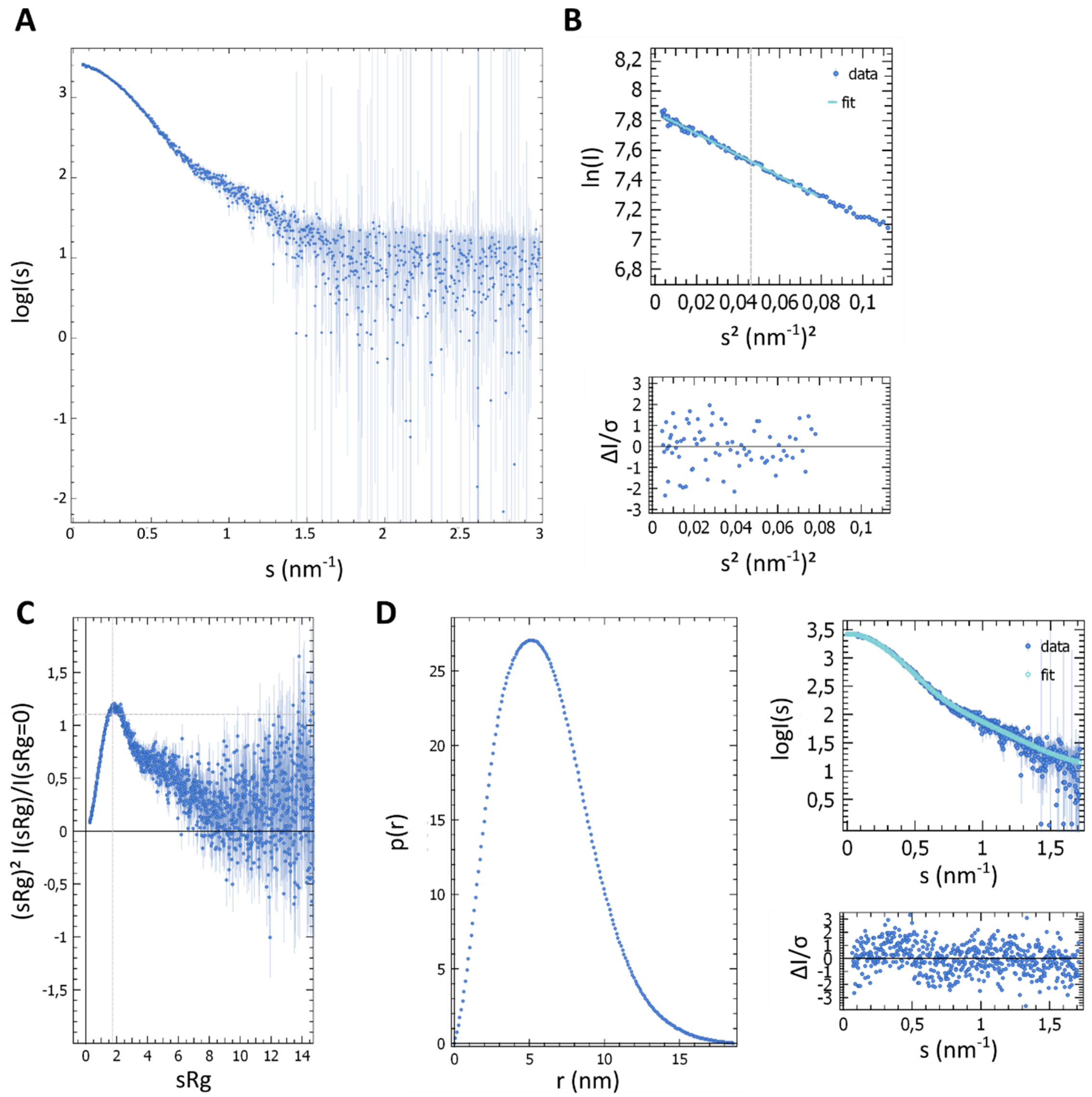

Figure S6 SEC-SAXS analysis of the purified Ybib-ObgE complex. **A)** Small angle X-ray scattering (SAXS) profile obtained for the YbiB-ObgE complex after buffer subtraction and averaging. **B)** Guinier plot with corresponding fit residuals. **C)** Dimensionless Kratky plot. **D)** *Left*: Pair distance distribution function (p(r)). *Right*: Fit of the p(r) function to the scattering profile and corresponding fit residuals. The SAXS data are also available in SASBDB under the accession code SASDQ78.

**peptide4-WW**	**peptide4-AA**	**peptide4-AW**	**peptide4-WA**
LEEIAEEDDED**W**DDD**W**DEDD	LEEIAEEDDED**A**DDD**A**DEDD	LEEIAEEDDED**A**DDD**W**DEDD	LEEIAEEDDED**W**DDD**A**DEDD

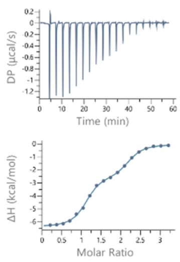
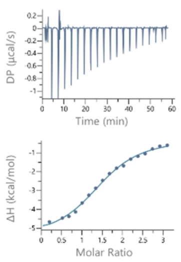
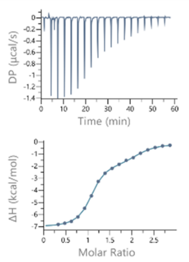
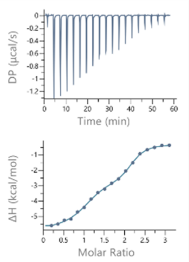

n_1 = 0.465 ± 0.002<br>K_{D1} = 35 ± 3 nM<br>n_2 = 0.554 ± 0.004<br>K_{D2} = 4.94 ± 0.05 µM	n = 0.66 ± 0.01<br>K_D = 10 ± 1 µM	n_1 = 0.472 ± 0.003<br>K_{D1} = 51 ± 4 nM<br>n_2 = 0.568 ± 0.004<br>K_{D2} = 4.02 ± 0.02 µM	n_1 = 0.471 ± 0.003<br>K_{D1} = 91 ± 11 nM<br>n_2 = 0.678 ± 0.008<br>K_{D2} = 16.3 ± 0.1 µM

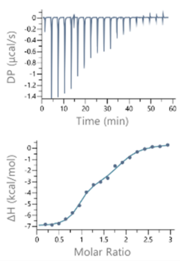
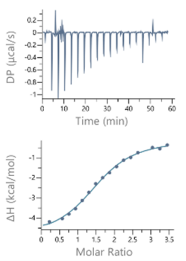
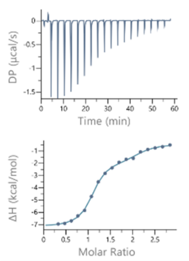
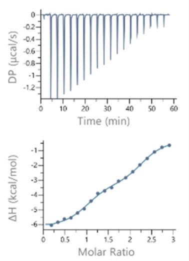

n_1 = 0.528 ± 0.008<br>K_{D1} = 75 ± 18 nM<br>n_2 = 0.68 ± 0.01<br>K_{D2} = 4.36 ± 0.08 µM	n = 0.632 ± 0.009<br>K_D = 10.0 ± 0.9 µM	n_1 = 0.476 ± 0.009<br>K_{D1} = 51 ± 10 nM<br>n_2 = 0.548 ± 0.009<br>K_{D2} = 4.29 ± 0.07 µM	n_1 = 0.434 ± 0.005<br>K_{D1} = 153 ± 20 nM<br>n_2 = 0.74 ± 0.03<br>K_{D2} = 16.1 ± 0.5 µM

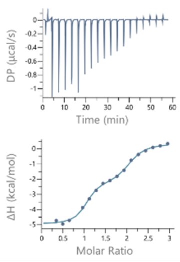
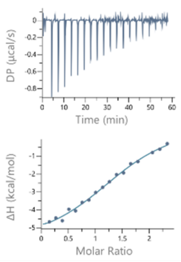
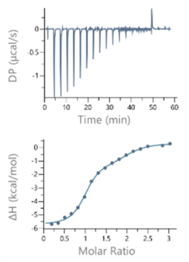
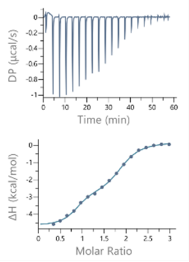

n_1 = 0.493 ± 0.006<br>K_{D1} = 33 ± 4 nM<br>n_2 = 0.580 ± 0.005<br>K_{D2} = 3.7 ± 0.1 µM	n = 0.64 ± 0.01<br>K_D = 15 ± 2 µM	n_1 = 0.49 ± 0.01<br>K_{D1} = 90 ± 26 nM<br>n_2 = 0.654 ± 0.02<br>K_{D2} = 5.8 ± 0.2 µM	n_1 = 0.528 ± 0.006<br>K_{D1} = 107 ± 18 nM<br>n_2 = 0.81 ± 0.01<br>K_{D2} = 8.8 ± 0.1 µM

Figure S7 ITC measurements to assess binding between YbiB and different variants of peptide4. The sample cell was filled with 75 µM peptide while the syringe was loaded with around 1.2 mM YbiB. Each measurement was performed in triplicate. The resulting binding isotherms were fitted on a suitable model to determine affinities (K_D) and stoichiometries (n).

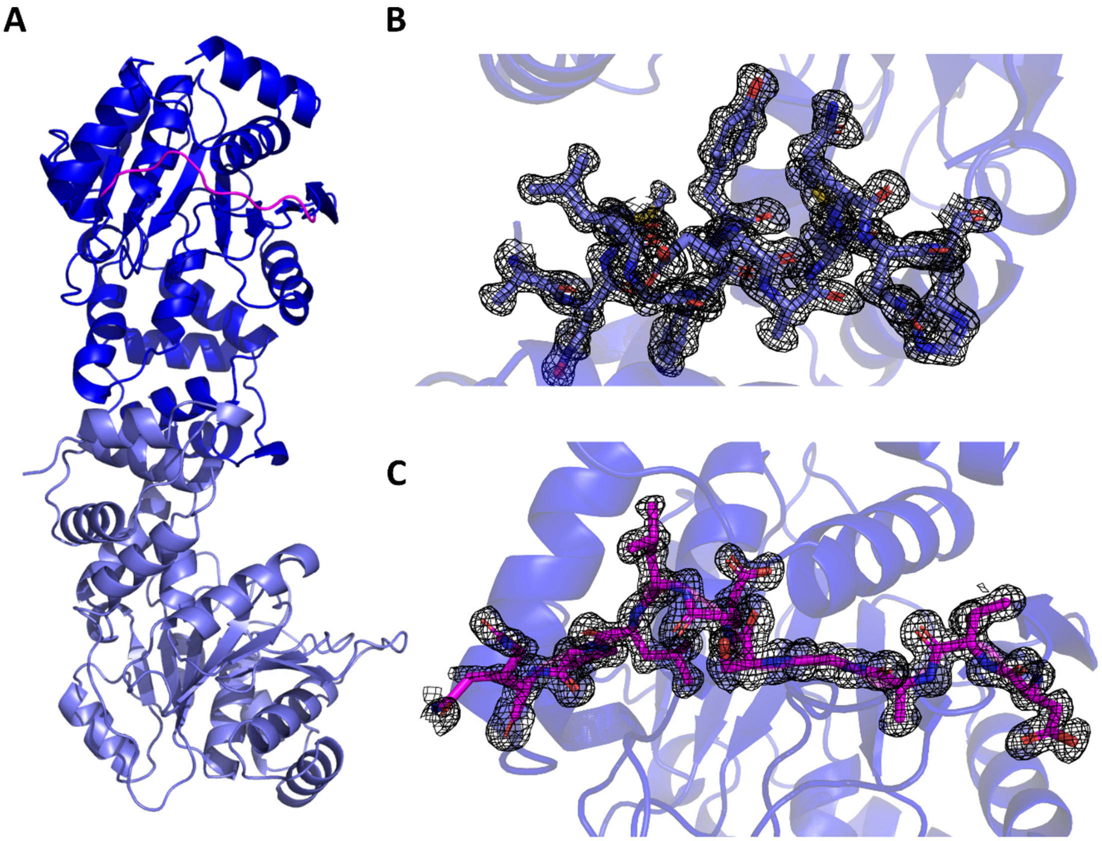

Figure S8 X-ray crystal structure obtained for the apo YbiB dimer (PDB: 8BFR). **A)** The structure of the apo YbiB dimer is solved to a resolution of 1.3 Å. The previously unresolved loop (residues 258 to 267) is indicated in magenta. The two protomers that make up the dimer are colored in blue and slate. **B)** Zoom-in picture of residues 57 to 69 that are part of an α-helix, displaying the electron density contoured at 1.5σ as a black mesh. **C)** Zoom-in picture of the previously missing loop (magenta), comprising residues 258 to 267. The electron density is contoured at 1σ and shown as a black mesh.

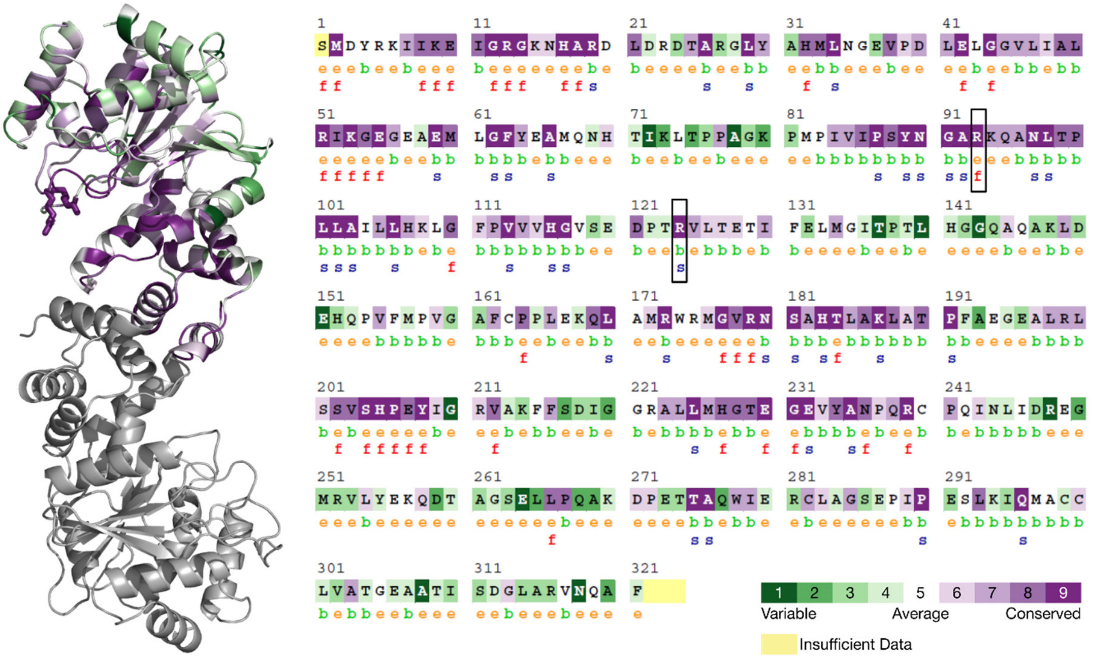

Figure S9 ConSurf analysis of the YbiB protein. *Left:* 3D representation of YbiB with residues of one protomer colored according to their degree of conservation, using the color-coded conservation scale. The conserved arginine residues Arg92 and Arg123 are presented in sticks. *Right:* Sequence representation of YbiB with residues colored according to their degree of conservation. The conserved arginine residues Arg92 and Arg123 are highlighted by boxes. Note that the residue numbering is shifted by 1, since the sequence includes a serine residue at position 1 that belongs to the N-terminal His$_6$-tag attached to the YbiB protein. e (orange): an exposed residue according to the NACCESS algorithm, b (green): a buried residue according to the NACCESS algorithm, f (red): a predicted functional residue (highly conserved and exposed), s (dark blue): a predicted structural residue (highly conserved and buried). Regions for which insufficient data was found are marked in yellow.

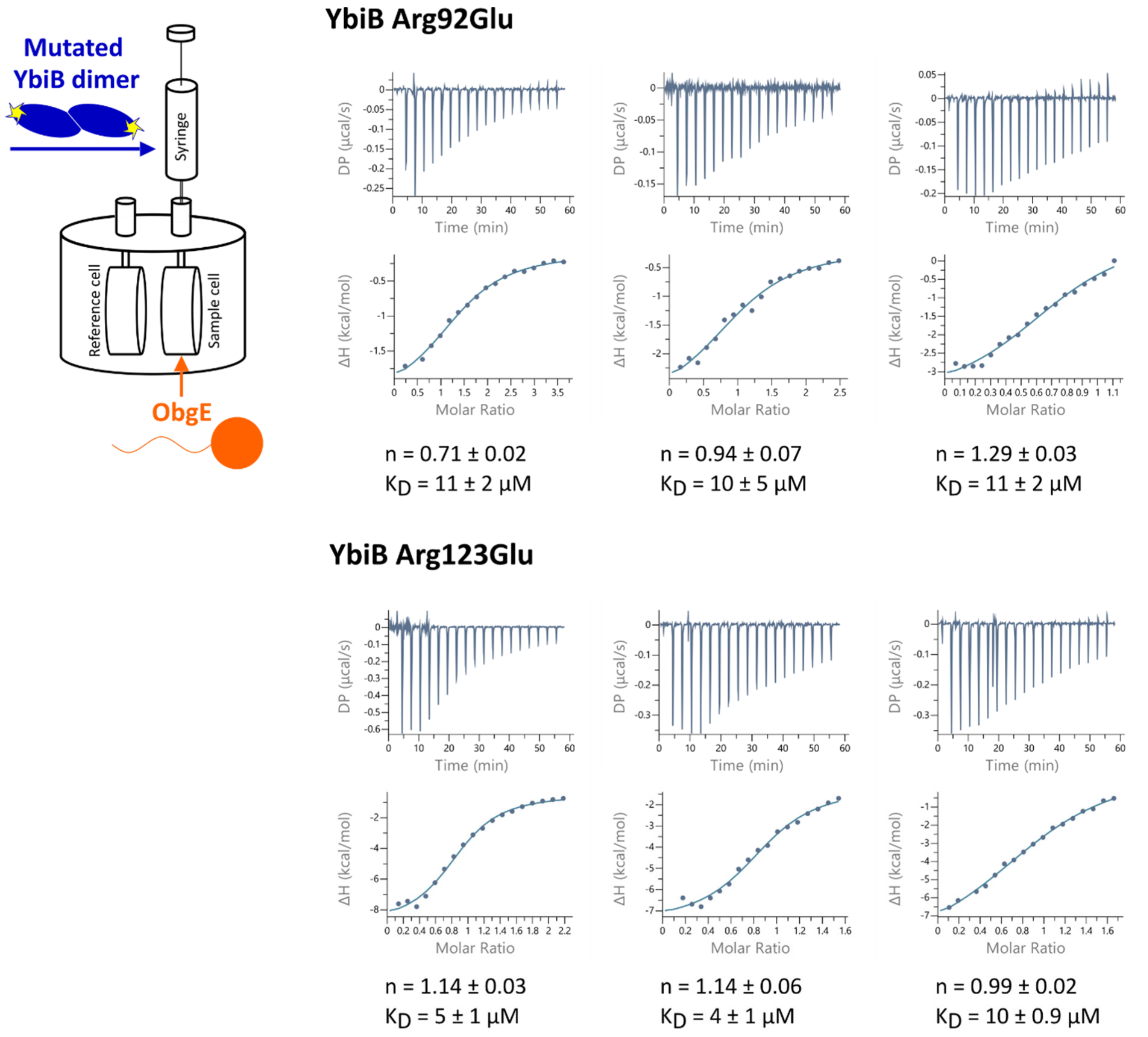

Figure S10 ITC measurements to assess binding between ObgE and two different arginine mutants of YbiB (Arg92Glu and Arg123Glu). The sample cell was filled with around 50 µM peptide, while the syringe was loaded with around 500 µM mutant YbiB. Each measurement was performed in triplicate. The resulting binding isotherms were fitted on a suitable model to determine affinities (K_D) and stoichiometries (n).

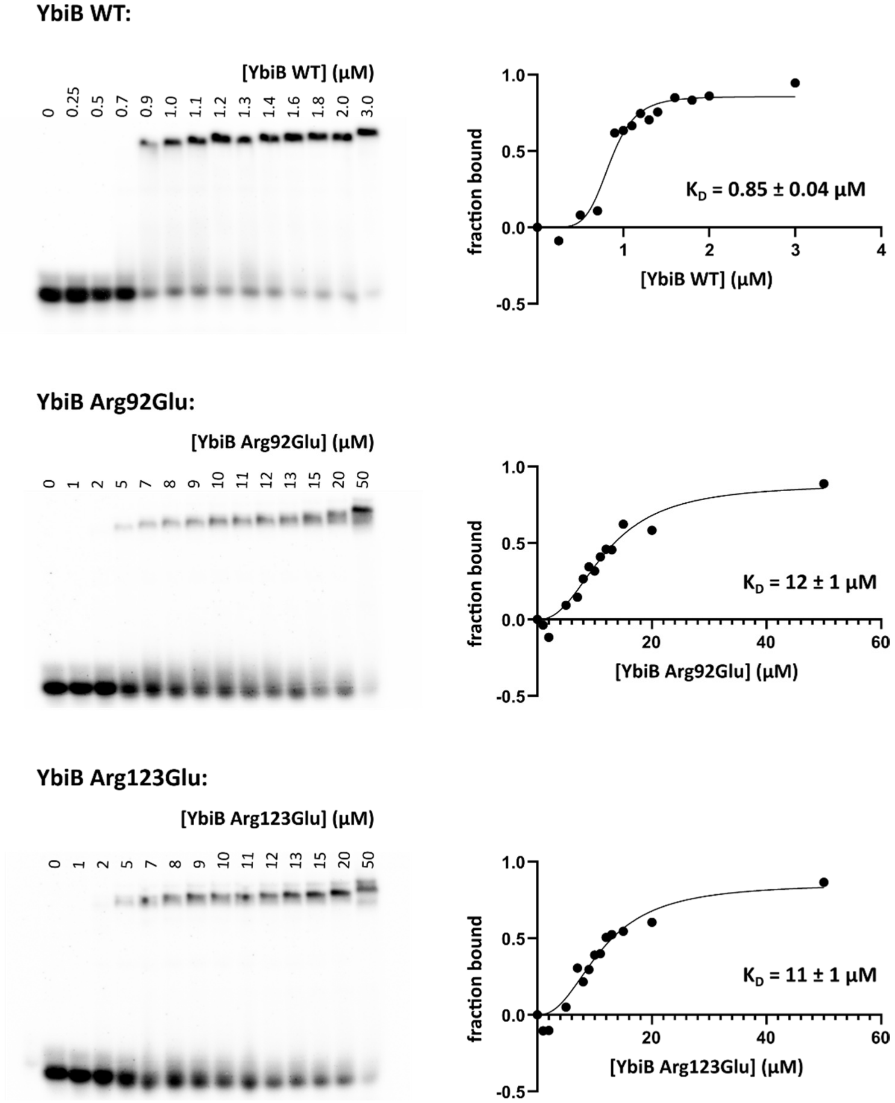

Figure S11 Electrophoretic mobility shift assays performed for YbiB WT and mutants. A ^{32}P-labeled ssDNA probe of 58 base pairs long was incubated with increasing concentrations of YbiB (WT or mutant). The samples were subsequently analyzed on a 6% polyacrylamide gel. Using the Image J software, the intensities of the signals of unbound DNA were determined in order to obtain the fractions of unbound DNA. Based on the unbound fractions, the fractions of bound DNA could be calculated and plotted against the YbiB (mutant) concentration. The obtained curves were fitted on the Hill equation to determine apparent K_D-values ± fitting error.

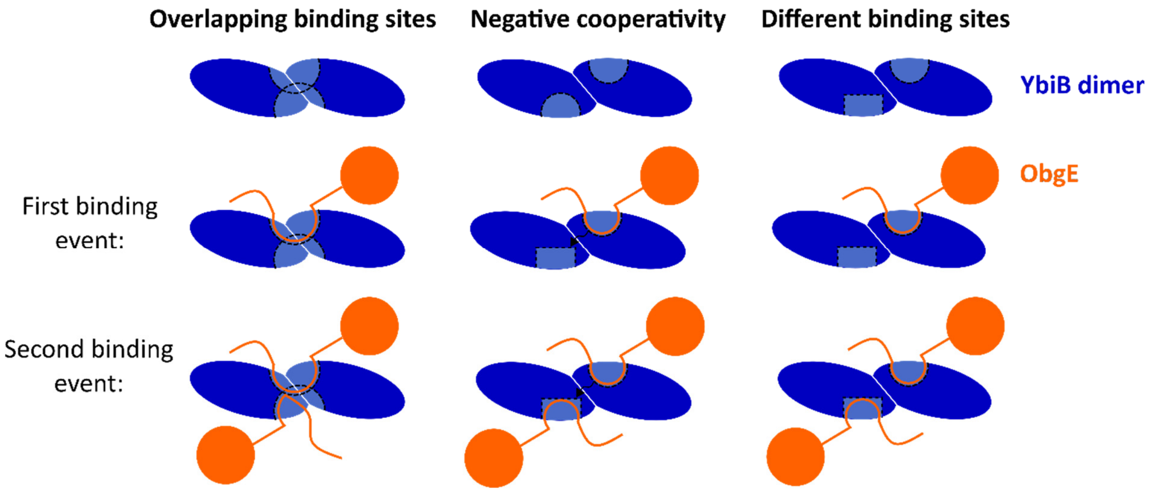

Figure S12 Possible theoretical scenarios to explain the observed biphasic interaction between YbiB and ObgE.

SUPPLEMENTARY TABLES

Table S1 Key reagents and biological resources used in this paper.

	Company name	Catalog number/Sequence/Reference/Genotype
**Enzymes**		
XbaI	New England Biolabs	R0145S
KpnI-HF	New England Biolabs	R3142S
EcoRI (FastDigest)	ThermoFisher Scientific	FD0274
XhoI (FastDigest)	ThermoFisher Scientific	FD0694
NheI (FastDigest)	ThermoFisher Scientific	FD0973
Alkaline Phosphatase grade I, from calf intestine	Roche	10108146001
T4 polynucleotide kinase	ThermoFisher Scientific	EK0031
**Kits**		
QuikChange Site-Directed Mutagenesis Kit	Stratagene	200518
NucleoSpin® Plasmid EasyPure	Filter Service (BIPP)	MN 740727.250
BACTH System Kit	Euromedex	EUK001
DNAeasy Blood Tissue!Kit	Qiagen	69504
Gibson Assembly	Bioké	E2611L
InFusion® HD Cloning Kit	Takara Bio	639650
**Non-standard chemicals**		
4-Benzoyl-L-phenylalanine (*p*Bpa)	Bachem	4017646.0005
Fmoc-protected amino acids (Fmoc-AA-OH)	Chem-Impex	
Fmoc-Arg(Pbf)-Wang	Advanced Chemtech	SR5109

Fmoc-Asp(tBu)-Wang	Advanced Chemtech	SD5110
Fmoc-Glu(tBu)-Wang	Advanced Chemtech	SE5120
Fmoc-Gln(Trt)-Wang	Advanced Chemtech	SQ5126
Rink amide-AM resin	Advanced Chemtech	SA6061
4-methylpiperidine	Sigma-Aldrich	P0445
N,N-dimethyl formamide (DMF)	Sigma-Aldrich	270547
N,N'-diisopropylcarbodiimide (DIC)	Fluorochem	132050
Ethyl cyano(hydroxyimino)acetate (Oxyma)	Fluorochem	043278
Acetic anhydride	Sigma-Aldrich	539996
Diisopropylethylamine (DIPEA)	Sigma-Aldrich	D125806
Dichloromethane (DCM)	Sigma-Aldrich	32222
Trifluoroacetic acid (TFA)	Fluorochem	008708
Triisopropylsilane (TIS)	Fluorochem	S17975
Dimethyl sulfoxide (DMSO)	Sigma-Aldrich	472301
Acetonitrile (used for peptide synthesis)	Sigma-Aldrich	34851
GTPγS	Jena Bioscience	NU-412-20
GDP	Sigma-Aldrich	G7127
GTP	Sigma-Aldrich	G8877
Tetra-n-butylammonium bromide	Sigma-Aldrich	8188390250
Acetonitrile (used for steady-state kinetics experiment)	Merck	1000302500
[γ-^{32}P]-ATP	PerkinElmer	NEG502A250UC
**Nucleotide-based reagents**		
Primers for introducing amber codons in ObgE and/or ObgE$_{D246G}$		
SPI-12019 (forward primer for ObgE$_{W122}$ and ObgE$_{D246G,W122}$)	IDT	5' GTTGCTAAGGGCGGCTAGCACGGT 3'
SPI-12020 (reverse primer for ObgE$_{W122}$ and ObgE$_{D246G,W122}$)	IDT	5' ATTGCCCAGACCGTGCTAGCCGCC 3'

SPI-10241 (forward primer for $ObgE_{I250}$)	IDT	5' CACCTCATCGATATCGATCCGTAGGACGGCACCGATCCGGTTG 3'
SPI-10242 (reverse primer for $ObgE_{I250}$)	IDT	5' CAACCGGATCGGTGCCGTCCTACGGATCGATATCGATGAGGTG 3'
SPI-11953 (forward primer for $ObgE_{D246G,I250}$)	IDT	5' GATCCGTAGGACGGCACCGAT 3'
SPI-11954 (reverse primer for $ObgE_{D246G,I250}$)	IDT	5' GCCGTCCTACGGATCGATACC 3'
SPI-10239 (forward primer for $ObgE_{Y269}$ and $ObgE_{D246G,Y269}$)	IDT	5' ATTATCAGCGAGCTGGAAAAATAGAGCCAGGATCTGGCGACGA 3'
SPI-10240 (reverse primer for $ObgE_{Y269}$ and $ObgE_{D246G,Y269}$)	IDT	5' TCGTCGCCAGATCCTGGCTCTATTTTTCCAGCTCGCTGATAAT 3'
SPI-11947 (forward primer for $ObgE_{Y388}$ and $ObgE_{D246G,Y388}$)	IDT	5' GAGTTCATTTAGAAGCGTTAA 3'
SPI-11948 (reverse primer for $ObgE_{Y388}$ and $ObgE_{D246G,Y388}$)	IDT	5' TTAACGCTTCTAAATGAACTCAAC 3'
SPI-10499 (forward primer for amplification of the MCS of pBAD/His A)	IDT	5' CTACTGTTTCTCCATACCCG 3'
SPI-10500 (reverse primer for amplification of the MCS of pBAD/His A)	IDT	5' TGTTTTATCAGACCGCTTCT 3'
Primers for B2H		
SPI-12423 (forward primer for amplification of *obgE* (XbaI restriction site))	IDT	5' CACCTCTAGAGAAGTTTGTTGATGAAGC 3'
SPI-12424 (reverse primer for amplification of *obgE* (KpnI restriction site))	IDT	5' ACTGGGTACCCGCTTGTAAATGAACTCAAC 3'
SPI-12671 (forward primer for amplification of *ybiB* (XbaI restriction site))	IDT	5' ACTGTCTAGAGGACTATCGCAAAATCATTAA 3'

SPI-12672 (reverse primer for amplification of *ybiB* (KpnI restriction site))	IDT	5’ CACCGGTACCGGAAATGCCTGATTAACGCGCG 3’
Primers for cloning *ybiB* in pBAD33Gm via Gibson assembly		
SPI-13850 (forward primer *ybiB*)	IDT	5’ GAGCTCAGGAGGAATTAACCATGGACTATCGCAAAATCATTAAAGAG 3’
SPI-13851 (reverse primer *ybiB*)	IDT	5’ CCGCCAAAACAGCCAAGCTTTTAAAATGCCTGATTAACGC 3’
SPI-13852 (forward primer pBAD33Gm)	IDT	5’ GCGTTAATCAGGCATTTTAAAAGCTTGGCTGTTTTGGCG 3’
SPI-13853 (reverse primer pBAD33Gm)	IDT	5’ GATTTTGCGATAGTCCATGGTTAATTCCTCCTGAGCTCGAATTCGCT 3’
Primers for deletion of *ybiB*		
SPI-12822 (forward primer)	Integrated DNA Technologies (IDT)	5’ TCGGCGGCGTTAATGATGTGAGTCAGGTAAGGAGTCGTAAGTGTAGGCTGGAGCTGCTTC 3’
SPI-12823 (reverse primer)	IDT	5’ TAAAAGCCGGATGACATGGCTCATCCGGCTGAGAAAAGAACATATGAATATCCTCCTTA 3’
Primers for cloning of Twin-Strep-tagged ObgE WT		
ObgETwinStrep_geneblock_FW	Sigma-Aldrich	5’ AGAGAAAGTCGAATTCATGTGG 3’
ObgETwinStrep_geneblock_RV	Sigma-Aldrich	5’ GGTGGTGGTGCTCGATTA 3’
Primers for cloning of His_6-tagged YbiB WT		
HisYbiB_FW	Sigma-Aldrich	5’ CTAGCTAGCATGGACTATCGCAAAATC 3’
HisYbiB_RV	Sigma-Aldrich	5’ CCGGAATTCTTATTAAAATGCCTGATTAACGCG 3’
Primers for site-directed mutagenesis of YbiB		
HisYbiBArg92Glu_FW	Sigma-Aldrich	5’ GGCGCGGAAAAACAGGCCAACC 3’
HisYbiBArg92Glu_RV	Sigma-Aldrich	5’ CTGTTTTTCCGCGCCGTTGTAACTG 3’
HisYbiBArg123Glu_FW	Sigma-Aldrich	5’ CCAACCGAAGTGCTGACTGAAACC 3’
HisYbiBArg123Glu_RV	Sigma-Aldrich	5’ CAGCACTTCGGTTGGATCTTCGC 3’
Double-stranded gene fragment used for cloning of Twin-Strep-tagged ObgE WT		
ObgETwinStrep_geneblock	Integrated DNA Technologies	5’ AGAGAAAGTCGAATTCATGTGGGATGATTATCATCGCCAGCAGCTTGAAGAGATTGCTG

		AAGAGGATGATGAAGACTGGGATGACGACTGGGACGAAGACGACGAAGAAGGCGTTGAG TTCATTTACAAGCGTAGCGCTTGGAGCCACCCGCAGTTCGAAAAAGGTGGTGGTAGCGGT GGTGGTAGCGGTGGTAGCGCATGGAGCCATCCGCAGTTTGAAAAATAATAACGAAAAATA ACTCGA 3’
DNA probe used for electrophoretic mobility shift assays		
EMSA_probe	Sigma-Aldrich	5’ GCTTTTGATGTACCAATGCATCACCATCACCATC AGGAAATGGGTAAGATGCTTGTAG 3’
**Peptides**		
Peptide1	In-house synthesis (Ballet lab)	H-EEAKQPEKVEFMWDDYHRQQLEEIAEEDDEDWDDDWDEDDEEGVEFIYKR-OH
Peptide2	In-house synthesis (Ballet lab)	H-EEAKQPEKVEFMWDDYHRQQ-OH
Peptide3	In-house synthesis (Ballet lab)	H-FMWDDYHRQQLEEIAEEDDE-OH
Peptide4	In-house synthesis (Ballet lab)	H-LEEIAEEDDEDWDDDWDEDD-OH
Peptide5	In-house synthesis (Ballet lab)	H-DWDDDWDEDDEEGVEFIYKR-OH
Peptide4-AA	In-house synthesis (Ballet lab)	H-LEEIAEEDDEDADDDADEDD-OH
Peptide4-AW	In-house synthesis (Ballet lab)	H-LEEIAEEDDEDADDDWDEDD-OH
Peptide4-WA	In-house synthesis (Ballet lab)	H-LEEIAEEDDEDWDDDADEDD-OH
**Organisms and strains**		
Escherichia coli DH10B (used for *in vivo* photocrosslinking experiments)		F$^-$, *mcrA,* Δ(*mrr-hsdRMS-mcrBC*), ϕ80*lacZ*ΔM15, Δ*lacX*74, *recA*1, *endA*1, *araD*139, Δ(*ara-leu*)7697, *galU*, *galK*, λ-, *rpsL*(Str^R), *nupG* Durfee *et al.*, 2008 (7) DOI: 10.1128/JB.01695-07#
Escherichia coli RH785 (used for B2H experiments)		F$^-$, λ-, *ilvG-*, *rfb*-50, *rph*-1 (MG1655), *cyaA*::frt Ronneau *et al.*, 2016 (8) DOI: 10.1038/ncomms11423

Escherichia coli BW25113 (used for experiments shown in Fig. 1)		F^-, Δ(*araD-araB*)567, Δ*lacZ*4787(::*rrnB*-3), λ-, *rph*-1, Δ(*rhaD-rhaB*)568, *hsdR*514<br>Datsenko and Wanner, 2000 (9)<br>DOI: 10.1073/pnas.120163297
Escherichia coli BL21 (DE3) pLysS (used for protein expression)		F^- *hsdS*$_B$ ($r_B^-m_B^-$) *gal dcm* (DE3) pLysS (Cm^R) ΔTonB<br>Weiner *et al.*, 1994 (10)
**Plasmid vectors**		
pBAD/His A-*obgE*		Verstraeten *et al.*, 2015 (1)<br>DOI: 10.1016/j.molcel.2015.05.011
pBAD/His A-*obgE*$_{D246G}$		Verstraeten *et al.*, 2019 (2)<br>DOI: 10.1111/mmi.14382
pSup-BpaRS-6TNR(D286R)		Ryu *et al.*, 2006 (3)<br>DOI: 10.1038/nmeth864
pUT18	Euromedex	EUK001 (BACTH System Kit)
pUT18C	Euromedex	EUK001 (BACTH System Kit)
pKT25	Euromedex	EUK001 (BACTH System Kit)
pKNT25	Euromedex	EUK001 (BACTH System Kit)
pKT25-*zip*		Karimova *et al.*, 1998 (11)<br>DOI: 10.1073/pnas.95.10.5752
pUT18-*zip*		Karimova *et al.*, 1998 (11)<br>DOI: 10.1073/pnas.95.10.5752
pUT18-*obgE*		This work
pUT18C-*obgE*		This work
pKT25-*obgE*		This work
pKNT25-*obgE*		This work
pUT18C-*ybiB*		This work
pUT18C-*ybiB*		This work
pKT25-*ybiB*		This work
pKNT25-*ybiB*		This work
pBAD33Gm		Dewachter *et al.*, 2017 (12)<br>DOI: 10.3389/fmicb.2017.01193
pBAD33Gm-*ybiB*		This work
pET28a	Novagen	69864

pET28a-*obgE*		Gkekas *et al*., 2017 (13)<br>DOI: 10.1074/jbc.M116.761809
pET28a-*obgE*$_{1-340}$		Gkekas *et al*., 2017 (13)<br>DOI: 10.1074/jbc.M116.761809
pET28a-*ybiB*		This work
pET28a-*ybiB*$_{R92E}$		This work
pET28a-*ybiB*$_{R123E}$		This work
pET22b	Novagen	69744
pET22b-*obgE* single Strep-tag		Gkekas *et al*., 2017 (13)<br>DOI: 10.1074/jbc.M116.761809
pET22b-*obgE* Twins-Strep-tag		This work

Table S2 Software programs and web servers used in this paper.

Program/Web Server	Reference/Source
MaxQuant (version 1.5.8.3)	Cox and Mann, 2008 (6)
Graphpad Prism (version 9.3.1)	Graphpad Software
Astra (version 7.3.0)	Wyatt Technology
MicroCal PEAQ ITC Analysis Software (version 1.30)	Malvern Panalytical
Refeyn AcquireMP (version 2022 R1)	Refeyn
Refeyn DiscoverMP (version 2022 R1)	Refeyn
PyMOL (version 2.3.2)	Schrödinger
SASFLOW	Franke *et al*., 2012 (14)<br>DOI: 10.1016/j.nima.2012.06.008
CHROMIXS	Panjkovich and Svergun, 2018 (15)<br>DOI: 10.1093/bioinformatics/btx846
ATSAS software package	Manalastas-Cantos *et al*., 2021 (16)<br>DOI : 10.1107/S1600576720013412
PRIMUS	Konarev *et al.*, 2003 (17)<br>DOI: 10.1107/S0021889803012779
AlphaFold-Multimer	Evans *et al*., 2021 (18)<br>DOI: 10.1101/2021.10.04.463034
CABS-dock web server	Kurcinski *et al*., 2015 (19)<br>DOI: 10.1093/nar/gkv456
ClusPro PeptiDock	Porter *et al*., 2017 (20)<br>DOI: 10.1093/bioinformatics/btx216
autoPROC	Vonrhein *et al*., 2011 (21)<br>DOI: 10.1107/S0907444911007773
STARANISO	Tickle *et al.*, 2018 (22)
Phenix suite	Liebschner *et al.*, 2019 (23)<br>DOI: 10.1107/S2059798319011471
Phaser	McCoy *et al*., 2007 (24)<br>DOI: 10.1107/S0021889807021206
Phenix.Refine	Afonine *et al*., 2012 (25)<br>DOI: 10.1107/S0907444912001308
Coot	Emsley *et al*., 2010 (26)<br>DOI: 10.1107/S0907444910007493
MolProbity	Williams *et al*., 2018 (27)<br>DOI: 10.1002/pro.3330
Consurf Web Server	Ashkenazy *et al*., 2016 (28)<br>DOI: 10.1093/nar/gkw408
ImageJ	Abràmoff *et al*., 2004 (29)

Table S3 Characteristics of the synthesized peptides used in this paper

Name	Lab code	Sequence	Bruto Formula	Mw (g/mol)	t_R [a] (min)	Calculated[b] Found		Yield (%)	Purity (%)
						Calculated	Found		
Peptide1	SBL-OBGE-01	H-EEAKQPEKVEFMWDDYHRQQLEEIAEEDDEDWDDDWDEDDEEGVEFIYKR-OH	$C_{275}H_{385}N_{67}O_{104}S$	6321.66	2.89	1265.5408 $[M+5H]^{5+}$	1265.5413 $[M+5H]^{5+}$	5	98
Peptide2	SBL-OBGE-05	H-EEAKQPEKVEFMWDDYHRQQ-OH	$C_{114}H_{165}N_{31}O_{37}S$	2592.17	2.63	1297.0930 $[M+2H]^{2+}$	1297.0900 $[M+2H]^{2+}$	12	>99
Peptide3	SBL-OBGE-04	H-FMWDDYHRQQLEEIAEEDDE-OH	$C_{112}H_{156}N_{28}O_{42}S$	2597.07	2.78	1299.5404 $[M+2H]^{2+}$	1299.5359 $[M+2H]^{2+}$	25	>99
Peptide4	SBL-ObgE-03	H-LEEIAEEDDEDWDDDWDEDD-OH	$C_{103}H_{136}N_{22}O_{51}$	2496.87	2.67	2497.8804 $[M+H]^{+}$	2497.8794 $[M+H]^{+}$	8	97
Peptide5	SBL-OBGE-02	H-DWDDDWDEDDEEGVEFIYKR-OH	$C_{113}H_{150}N_{26}O_{44}$	2575.03	2.82	1288.5228 $[M+2H]^{2+}$	1288.5244 $[M+2H]^{2+}$	20	>99
Peptide4-AA	SBL-OBGE-06	H-LEEIAEEDDEDADDDADEDD-OH	$C_{87}H_{126}N_{20}O_{51}$	2266.79	2.23	2267.7959 $[M+H]^{+}$	2267.7786 $[M+H]^{+}$	18	>99
Peptide4-WA	SBL-OBGE-10	H-LEEIAEEDDEDWDDDADEDD-OH	$C_{95}H_{131}N_{21}O_{51}$	2381.83	2.47	1202.9139 $[M+H+Na]^{2+}$	1202.9662 $[M+H+Na]^{2+}$	6	>99
Peptide4-AW	SBL-OBGE-11	H-LEEIAEEDDEDADDDWDEDD-OH	$C_{95}H_{131}N_{21}O_{51}$	2381.83	2.46	2382.8381 $[M+H]^{+}$	2382.8516 $[M+H]^{+}$	2	>99

[a] HPLC analysis was performed on a Hitachi Chromaster system (Chromaster HPLC 5260 autosampler, Chromaster HPLC 5160 Pump, Chromaster HPLC 5310 column and a Chromaster HPLC 5430 diode array detector). The mobile phase consists of 0.1% TFA in AcN and 0.1% TFA in Milli-Q water. The analyzed peptides eluted through a Chromolith® High Resolution C18 end-capped column (50 mm x 4.6 mm, 1.1 µm, 150 Å) using a gradient from 1% to 100 % of AcN over 6 min at a flow rate of 2.8 mL min^{-1} .

[b] HRMS analysis was performed on a Waters 600 HPLC (combined with a Waters 2487 UV detector at 215 nm) connected to a Micromass QTOF-micro system. The mobile phase consists of 0.1% FA in AcN and 0.1% FA in Milli-Q water The analyzed peptides eluted through an EC NUCLEODUR C18 endcapped column (150 mm x 2 mm, 5 µm, 300 Å), using a gradient from 3% to 100% AcN over 20 min with a flow rate of 0.3 mL/min. A Reserpine (2.10^{-3} mg/ml) solution in H_2O/ACN (50:50% v/v) was utilized as reference for the HRMS.

Table S4 Parameters for the SEC-SAXS analysis of the purified YbiB-ObgE complex

**Data collection parameters**	
Beamline	P12, Petra III
Detector	Pilatus 6M
Wavelength (Å)	1.24
Detector distance (m)	3
s range (nm^{-1})[a]	0.05 - 7.4
Exposure time	Continuous 1 s data measurements of SEC elution
SEC column	Superdex 200 10/300
Loading concentration (mg/ml)	7.6
Injection volume (µl)	50
Flow rate (ml/min)	0.6
Temperature	25°C
**Structural parameters**	
I(0) (from p(r))	2604 ± 8
Rg (nm) (from p(r))	4.80 ± 0.03
I(0) (from Guinier)	2581 ± 8
Rg (nm) (from Guinier)	4.65 ± 0.02
Dmax (nm)	18.62
**Molar mass determination (kDa)**	
Based on the Porod invariant	179.7
Based on the Fischer method	152.1
Based on the correlation volume	167.5
Based on the “Size and Shape” machine learning method	174.8
Based on the Bayesian inference approach	169.6 with credibility interval [151.5; 176.6]
From sequence (including tags and assuming a 1:1 stochiometry)	165.9

[a]Momentum transfer $|s| = 4\pi\sin(\theta)/\lambda$ with λ the wavelength of the X-rays and 2θ the scattering angle

Table S5 X-ray data collection and refinement statistics

	apo YbiB	YbiB in complex with ObgE C-terminal peptide4
PDB number	8BFR	8BFT
**Data collection**		
Synchrotron	Soleil	Soleil
Beamline	PROXIMA-1	PROXIMA-1
Wavelength (Å)	0.97856	0.97856
Resolution range (Å)[a]	66.55 – 1.3 (1.347-1.3)	47.03 – 1.192 (1.235-1.192)
Space group	C121	C121
Unit cell dimensions (Å)	a = 134.154	a = 133.469
	b = 64.004	b = 63.895
	c = 47.496	c = 47.416
Unit cell angles (°)	α = 90.000°	α = 90.000°
	β = 97.218°	β = 97.353°
	γ = 90.000°	γ = 90.000°
Spherical completeness (%)[a]	82.2 (21.2)	69.6 (12.4)
Ellipsoidal completeness (%)[a]	93.6 (50.2)	92.8 (57.1)
Unique reflections	96074	123740
Mean(I)/σ(I)[a]	10.8 (1.7)	9.4 (1.6)
CC(1/2)[a]	0.998 (0.624)	0.997 (0.696)
Multiplicity[a]	6.8 (5.6)	6.8 (5.8)
R_{meas} (all I+ & I-) (%)[a]	9.5 (106.8)	9.5 (103.2)
**Refinement**		
Resolution range (Å)[a]	66.55 – 1.3 (1.347-1.3)	47.03 – 1.192 (1.235-1.192)
R_{work} (%)[a]	13.53 (27.15)	13.69 (22.62)
R_{free} (%)[a, b]	16.67 (32.34)	16.68 (30.10)
Model content		
Molecules per ASU	1	1
Protein atoms per ASU	2469	2530
Ligand atoms per ASU	28	34
Water molecules per ASU	413	386
Wilson B factors (Å²)	12.62	13.81
Average B factors (Å²)	21.61	22.78
Protein atoms	18.38	20.08
Ligand atoms	75.42	72.61
Water molecules	39.34	38.66
Rmsd bonds (Å)	0.009	0.009
Rmsd angles (°)	1.09	1.05
Ramachandran plot (%) (favored, outliers)	98.75, 0.00	98.76, 0.00

[a] Values in parentheses are for the highest-resolution shell

[b] R_{free} is based on a subset of 5% of reflections omitted during refinement

ASU: asymmetrical unit

Table S6 Thermodynamic parameters obtained from the ITC measurements performed with YbiB WT in the syringe and ObgE or derived peptides in the sample cell. In case measurements were performed in triplicate, the parameters for each individual repeat are given.

Sample in sample cell	First binding event			Second binding event		
	ΔH (kcal/mol)	-TΔS (kcal/mol)	ΔG (kcal/mol)	ΔH (kcal/mol)	-TΔS (kcal/mol)	ΔG (kcal/mol)
ObgE	-6.78 ± 0.08	-4.11	-10.89	-3.42 ± 0.2	-3.62	-7.04
	-7.93 ± 0.8	-2.95	-10.88	-3.29 ± 0.1	-4.00	-7.29
	-8.13 ± 0.08	-2.26	-10.39	-2.27 ± 0.07	-5.10	-7.37
Peptide1	-10.70 ± 0.08	1.45	-9.25	-1.57 ± 0.07	-5.46	-7.03
	-13.6 ± 0.3	3.99	-9.6	-1.35 ± 0.1	-5.98	-7.33
	-13.0 ± 0.2	3.70	-9.3	-1.17 ± 0.2	-5.98	-7.15
Peptide2	NBD	NBD	NBD			
Peptide3	-5.65 ± 0.04	-1.46	-7.11			
Peptide4	-9.45 ± 0.05	-0.721	-10.17	-3.63 ± 0.05	-3.61	-7.24
	-10.3 ± 0.2	0.581	-9.7	-2.97 ± 0.2	-4.34	-7.31
	-7.90 ± 0.08	-2.32	-10.22	-2.46 ± 0.09	-4.95	-7.41
Peptide4-AW	-8.94 ± 0.03	-1.01	-9.95	-4.78 ± 0.05	-2.58	-7.36
	-8.53 ± 0.06	-1.41	-9.94	-4.93 ± 0.2	-2.40	-7.33
	-7.4 ± 0.1	-2.19	-9.59	-3.78 ± 0.2	-3.36	-7.14
Peptide4-WA	-8.22 ± 0.05	-1.39	-9.61	-2.62 ± 0.07	-3.91	-6.53
	-9.88 ± 0.08	0.582	-9.30	-2.4 ± 0.2	-4.10	-6.5
	-7.16 ± 0.08	-2.34	-9.50	-1.30 ± 0.09	-5.60	-6.90
Peptide4-AA	-8.0 ± 0.1	1.22	-6.78			
	-7.7 ± 0.1	0.874	-6.83			
	-10.7 ± 0.3	4.10	-6.6			
Peptide5	-4.38 ± 0.06	-4.00	-8.38			

NBD: No binding detected

SUPPLEMENTARY REFERENCES

1. Verstraeten, N., Knapen, W.J., Kint, C.I., Liebens, V., Van den Bergh, B., Dewachter, L., Michiels, J.E., Fu, Q., David, C.C., Fierro, A.C. *et al.* (2015) Obg and membrane depolarization are part of a microbial bet-hedging strategy that leads to antibiotic tolerance. Mol. Cell, 59, 9-21.
2. Verstraeten, N., Gkekas, S., Kint, C.I., Deckers, B., Van den Bergh, B., Herpels, P., Louwagie, E., Knapen, W., Wilmaerts, D., Dewachter, L., Fauvart, M., Singh, R.K., Michiels, J. and Versées, W. (2019) Biochemical determinants of ObgE-mediated persistence. Mol. Microbiol., 112, 1593-1608.
3. Ryu, Y. and Schultz, P.G. (2006) Efficient incorporation of unnatural amino acids into proteins in *Escherichia coli*. Nat. Methods, 3, 263-265.
4. Shevchenko, A., Wilm, M., Vorm, O. and Mann, M. (1996) Mass spectrometric sequencing of proteins silver-stained polyacrylamide gels. Anal Chem, 68, 850-858.
5. Candiano, G., Bruschi, M., Musante, L., Santucci, L., Ghiggeri, G.M., Carnemolla, B., Orecchia, P., Zardi, L. and Righetti, P.G. (2004) Blue silver: a very sensitive colloidal Coomassie G-250 staining for proteome analysis. Electrophoresis, 25, 1327-1333.
6. Cox, J. and Mann, M. (2008) MaxQuant enables high peptide identification rates, individualized ppb-range mass accuracies and proteome-wide protein quantification. Nat. Biotechnol., 26, 1367-1372.
7. Durfee, T., Nelson, R., Baldwin, S., Plunkett, G., 3rd, Burland, V., Mau, B., Petrosino, J.F., Qin, X., Muzny, D.M., Ayele, M. *et al.* (2008) The complete genome sequence of *Escherichia coli* DH10B: insights into the biology of a laboratory workhorse. J Bacteriol, 190, 2597-2606.
8. Ronneau, S., Petit, K., De Bolle, X. and Hallez, R. (2016) Phosphotransferase-dependent accumulation of (p) ppGpp in response to glutamine deprivation in *Caulobacter crescentus*. Nat. Commun., 7, 1-12.
9. Datsenko, K.A. and Wanner, B.L. (2000) One-step inactivation of chromosomal genes in *Escherichia coli* K-12 using PCR products. Proc. Natl. Acad. Sci. U. S. A., 97, 6640-6645.
10. Weiner, M., Anderson, C., Jerpseth, B., Wells, S., Johnson-Browne, B. and Vaillancourt, P. (1994) Studier pET system vectors and hosts. Strateg. Mol. Biol., 7, 41-43.
11. Karimova, G., Pidoux, J., Ullmann, A. and Ladant, D. (1998) A bacterial two-hybrid system based on a reconstituted signal transduction pathway. Proc. Natl. Acad. Sci. U. S. A., 95, 5752-5756.
12. Dewachter, L., Verstraeten, N., Jennes, M., Verbeelen, T., Biboy, J., Monteyne, D., Pérez-Morga, D., Verstrepen, K.J., Vollmer, W., Fauvart, M. and Michiels, J. (2017) A mutant isoform of ObgE causes cell death by interfering with cell division. Front. Microbiol., 8, 1193.
13. Gkekas, S., Singh, R.K., Shkumatov, A.V., Messens, J., Fauvart, M., Verstraeten, N., Michiels, J. and Versées, W. (2017) Structural and biochemical analysis of *Escherichia coli* ObgE, a central regulator of bacterial persistence. J. Biol. Chem., 292, 5871-5883.
14. Franke, D., Kikhney, A.G. and Svergun, D.I. (2012) Automated acquisition and analysis of small angle X-ray scattering data. Nucl. Instrum. Methods Phys. Res., Sect. A, 689, 52-59.
15. Panjkovich, A. and Svergun, D.I. (2018) CHROMIXS: automatic and interactive analysis of chromatography-coupled small-angle X-ray scattering data. Bioinformatics, 34, 1944-1946.
16. Manalastas-Cantos, K., Konarev, P.V., Hajizadeh, N.R., Kikhney, A.G., Petoukhov, M.V., Molodenskiy, D.S., Panjkovich, A., Mertens, H.D., Gruzinov, A., Borges, C., Jeffries, C.M., Svergun, D.I. and Franke, D. (2021) ATSAS 3.0: expanded functionality and new tools for small-angle scattering data analysis. J. Appl. Crystallogr., 54, 343-355.

17. Konarev, P.V., Volkov, V.V., Sokolova, A.V., Koch, M.H. and Svergun, D.I. (2003) PRIMUS: a Windows PC-based system for small-angle scattering data analysis. J. Appl. Crystallogr., 36, 1277-1282.
18. Evans, R., O'Neill, M., Pritzel, A., Antropova, N., Senior, A.W., Green, T., Žídek, A., Bates, R., Blackwell, S., Yim, J. *et al.* (2021) Protein complex prediction with AlphaFold-Multimer. BioRxiv.
19. Kurcinski, M., Jamroz, M., Blaszczyk, M., Kolinski, A. and Kmiecik, S. (2015) CABS-dock web server for the flexible docking of peptides to proteins without prior knowledge of the binding site. Nucleic Acids Res., 43, W419-W424.
20. Porter, K.A., Xia, B., Beglov, D., Bohnuud, T., Alam, N., Schueler-Furman, O. and Kozakov, D. (2017) ClusPro PeptiDock: efficient global docking of peptide recognition motifs using FFT. Bioinformatics, 33, 3299-3301.
21. Vonrhein, C., Flensburg, C., Keller, P., Sharff, A., Smart, O., Paciorek, W., Womack, T. and Bricogne, G. (2011) Data processing and analysis with the autoPROC toolbox. Acta Crystallogr., Sect. D: Biol. Crystallogr., 67, 293-302.
22. Tickle, I.J., Flensburg, C., Keller, P., Paciorek, W., Sharff, A., Vonrhein, C., Bricogne, G. (2018). Global Phasing Ltd., Cambridge, United Kingdom.
23. Liebschner, D., Afonine, P.V., Baker, M.L., Bunkóczi, G., Chen, V.B., Croll, T.I., Hintze, B., Hung, L.-W., Jain, S., McCoy, A.J. *et al.* (2019) Macromolecular structure determination using X-rays, neutrons and electrons: recent developments in Phenix. Acta Crystallogr., Sect. D: Struct. Biol., 75, 861-877.
24. McCoy, A.J., Grosse-Kunstleve, R.W., Adams, P.D., Winn, M.D., Storoni, L.C. and Read, R.J. (2007) Phaser crystallographic software. J. Appl. Crystallogr., 40, 658-674.
25. Afonine, P.V., Grosse-Kunstleve, R.W., Echols, N., Headd, J.J., Moriarty, N.W., Mustyakimov, M., Terwilliger, T.C., Urzhumtsev, A., Zwart, P.H. and Adams, P.D. (2012) Towards automated crystallographic structure refinement with phenix. refine. Acta Crystallogr., Sect. D: Biol. Crystallogr., 68, 352-367.
26. Emsley, P., Lohkamp, B., Scott, W.G. and Cowtan, K. (2010) Features and development of Coot. Acta Crystallogr., Sect. D: Biol. Crystallogr., 66, 486-501.
27. Williams, C.J., Headd, J.J., Moriarty, N.W., Prisant, M.G., Videau, L.L., Deis, L.N., Verma, V., Keedy, D.A., Hintze, B.J., Chen, V.B. *et al.* (2018) MolProbity: More and better reference data for improved all-atom structure validation. Protein Sci., 27, 293-315.
28. Ashkenazy, H., Abadi, S., Martz, E., Chay, O., Mayrose, I., Pupko, T. and Ben-Tal, N. (2016) ConSurf 2016: an improved methodology to estimate and visualize evolutionary conservation in macromolecules. Nucleic Acids Res., 44, W344-W350.
29. Abràmoff, M.D., Magalhães, P.J. and Ram, S.J. (2004) Image processing with ImageJ. Biophotonics Int., 11, 36-42.
